# Quality indicators for the management of clinical simulation centers and laboratories: a scoping review

**DOI:** 10.1590/1518-8345.8215.4906

**Published:** 2026-08-11

**Authors:** Isaac Sebastião Nunes Santos, Cláudio José de Souza, Rodrigo Leite Hipolito, Márcia Farias de Oliveira, Carolina Baptista Ribeiro, André da Silva Brites

**Affiliations:** 1 Universidade Federal Fluminense, Escola de Enfermagem Aurora de Afonso Costa, Niterói, RJ, Brazil.; 2 Hospital Universitário Antônio Pedro, Centro de Terapia Intensiva, Niterói, RJ, Brazil.

**Keywords:** Simulation Training, Management Indicators, Quality Indicators, Health Care, Quality Assurance, Health Care, Organization and Administration, Health Management

## Abstract

**Objective:**

to map quality indicators for the management of clinical simulation centers and laboratories in the literature.

**Method:**

scoping review conducted according to JBI guidelines and the Preferred Reporting Items for Systematic Reviews and Meta-Analyses for Scoping Reviews extension. Searches were conducted for quality indicators for the management of clinical simulation centers/laboratories published since 1990, across four information sources and on Google Scholar, without restriction on method, language, or origin. The data were extracted after thematic grouping and categorized according to the Structure, Process, and Outcome triad recommended by Donabedian, and correlated with the best-practice standards of the International Nursing Association for Clinical Simulation and Learning.

**Results:**

of the six studies selected between 2013 and 2024, eighty-four quality indicators were mapped. Despite the high number of indicators, they are not fully applicable to the evaluation of management processes in clinical simulation centers/laboratories. Planning, resource organization, staff training, supply management, equipment maintenance, and continuous monitoring remain underdeveloped in the literature.

**Conclusion:**

it is recommended that future research propose and validate indicators of management processes in clinical simulation centers/laboratories, considering their particularities and operational challenges, with the aim of continuously improving teaching and learning spaces in health.

## Highlights

**(1)** 84 quality indicators identified for the management of clinical simulation centers. **(2)** Evaluation indicators are still not very applicable to management processes. **(3)** Predominance of pedagogical indicators over managerial ones. **(4)** Subsidies for structuring specific indicators for managerial evaluation. **(5)** Need for validation of managerial indicators in clinical simulation centers.

## Introduction

Clinical simulation has established itself as a tool for teaching and training students/health professionals. It is a technique that creates a simulated situation or environment to enable health professionals to experience a representation of a real event, supporting practice, learning, evaluation, testing, or understanding of systems or human actions. In addition to the direct benefits for the education and training of students/health professionals, clinical simulation also enables the optimization of health systems, improves the care provided and contributes to patient safety^1^.

The use of simulation in health education began in the 18th century, with anatomical mannequins used to teach obstetrics in France; however, it gained greater momentum in the 1950s with the development of low- and high-fidelity electronic simulators capable of reproducing physiological functions, responding to medications, and teaching basic airway management^2^ ^-^ ^3^. In subsequent decades, technological advances led to increasingly sophisticated simulators, transforming health education and spurring the creation of simulation centers and laboratories; however, the high cost of specialized equipment and software remains a challenge for institutions with limited resources^4^.

Given this scenario, it is necessary to consider that implementing a simulation program requires a significant investment in resources, personnel, and time. This must be recognized and carefully considered before the start of the project through a cost-effectiveness analysis based on potential benefits in clinical practice and patient outcomes. Thus, effectiveness alone does not justify high investments, and it is essential also to analyze efficiency, that is, how much value is generated in relation to the cost incurred^5^.

Given the expansion of clinical simulation centers/laboratories in educational institutions and health services, it is essential that these environments be managed effectively to ensure their relevance in the educational process and their positive impact on professional training. To this end, it is essential that the management of these spaces incorporate, among other strategies, the development and use of quality indicators to monitor and evaluate their functioning, performance, and contribution to the improvement of teaching^6^.

The use of quality indicators has become common practice in the administration of health services, as it enables both the evaluation of results and the continuous improvement of pedagogical and operational activities^7^. In the context of simulation centers/laboratories, it is important that these indicators encompass elements related to structure, processes, and results, including available infrastructure, instructor training, adopted methodologies, and observed effects on student/health professional learning.

Quality indicators, whether qualitative or quantitative, are essential in supporting strategic decision-making. Their selection and use must be carefully planned, considering the specific characteristics of clinical simulation centers and laboratories, accreditation requirements, and previously defined strategic objectives^8^.

It is believed that the increasing adoption of clinical simulation centers/laboratories requires efficient, evidence-based management. However, the literature lacks studies that address specific indicators of the management processes in these environments. The absence of these parameters compromises the evaluation of efficiency and continuous improvement. This study is justified by the need to map quality indicators for the management of clinical simulation centers/laboratories, thereby contributing to their sustainability, strategic alignment, and the qualification of educational practices. Given this context, the objective of this study was to map quality indicators for the management of clinical simulation centers and laboratories in the literature.

## Method

### Type of review 

This scoping review follows the guidelines and recommendations of the JBI manual and the guidelines of the Preferred Reporting Items for Systematic Reviews and Meta-Analyses Extension for Scoping Reviews (PRISMA-ScR)^9^ ^-^ ^10^.

This review format enables mapping and gathering a wide range of materials related to a specific topic, providing a broad understanding of the subject. It also allows consideration of studies with varying levels of evidence and methods, including information sources and gray literature. However, it is not intended to perform critical evaluation, bias risk analysis, or evidence-level ranking^9^.

To ensure the reliability and fairness of the review process, the protocol was registered in the Open Science Framework (https://doi.org/10.17605/OSF.IO/6AGBE).

### Eligibility criteria

This review included original studies that addressed quality indicators for the management of clinical simulation centers/laboratories. Studies published since 1990 were considered, as this period marked the greatest development in clinical simulation. There were no restrictions on the methodological design, language, or country of origin of the studies, and publications that addressed the research question and met the inclusion criteria defined using the Population-Context-Concept (PCC) mnemonic strategy applied in scope reviews were included. Studies that did not adhere to the theme, did not fully meet the established PCC components, or contained duplicate publications across databases were excluded.

According to the JBI manual guidelines, the PCC mnemonic should follow the relevant reference standard (Population, Concept, and Context) to define a clear title, and this definition must appear verbatim in the objective, research question, and inclusion criteria. In this study, Population = clinical simulation centers/laboratories; Concept = quality/management indicators; Context = management and monitoring of these centers. Thus, the review established the following question: what quality indicators are necessary for the management of clinical simulation centers/laboratories?

### Sources of information

The search for articles was conducted in scientific literature sources, including the Medical Literature Analysis and Retrieval System Online (MEDLINE) via PubMed, the Cumulative Index to Nursing and Allied Health Literature (CINAHL), the Latin American and Caribbean Health Sciences Literature (LILACS), and Scopus. In addition, Google Scholar was used to search for theses and dissertations (gray literature) and to compile the reference list.

### Search strategies

The search strategies were developed by the lead author, in collaboration with his advisor and with support from a librarian specializing in constructing search strategies for scoping reviews. To ensure the adequacy of the terms and sources, a preliminary consultation was conducted on August 15, 2024.

The search strategy was conducted by combining descriptors and free-text synonyms corresponding to the elements of the acronym PCC to identify scientific evidence addressing the study’s research question. For term selection, controlled health vocabularies were consulted, including Descriptors in Health Sciences- DeCS and Medical Subject Headings- MeSH.

Works published since 1990 were included, as this period marked the greatest development in clinical simulation. No filters were applied regarding language or study design, ensuring a broad and comprehensive search. In addition, search strategies were developed in accordance with the Peer Review of Electronic Search Strategies (PRESS) guidelines, thereby ensuring the quality and rigor of the process.

The search strategies were developed from a preliminary search, followed by an analysis of the titles, abstracts, and keywords of the retrieved studies, which enabled the refinement and selection of terms related to health quality indicators and simulation-based training. To answer the review question, combinations of the descriptors “Quality Indicators, Health Care” and “Quality Assurance, Health Care” were used, as well as free terms and controlled descriptors associated with clinical simulation and simulation training. The searches were structured using Boolean operators and adapted to the technical specificities of each database, including linguistic variations when relevant, as shown in Figure 1.

**Figure 1:** Search strategies in the databases searched. Niterói, RJ, Brazil, 2025

**Search strategies**	
**PubMed**	(Quality Indicators, Health Care[mj] OR Quality Assurance, Health Care[mj] OR Quality Indicator*[tiab] OR Health Metric*[tiab] OR Quality Metric*[tiab] OR Quality Assessment[tiab] OR Quality Assurance[tiab] OR Indicator*[ti] OR Metric*[ti]) AND (Simulation Training[mj] OR Simulation Training[tiab] OR Simulation-based Training[tiab] OR Realistic Simulat*[tiab] OR Interactive Training[tiab] OR Interactive Learning[tiab] OR Interactive Simulat*[tiab] OR Clinical Simulat*[tiab] OR Healthcare Simulat*[tiab] OR Patient Simulat*[tiab])
**Scopus**	TITLE-ABS(“Quality Indicator*” OR “Health Metric*” OR “Quality Metric*” OR “Quality Assessment” OR “Quality Assurance” OR Indicator* OR Metric*) AND TITLE(“Simulation Training” OR “Simulation-based Training” OR “Realistic Simulation” OR “Interactive Training” OR “Interactive Learning” OR “Interactive Simulation” OR “Clinical Simulation” OR “Healthcare Simulation” OR “Patient Simulation”)
**CINAHL**	(“Quality Indicators” OR “Health Metrics” OR “Quality Metrics” OR “Quality Assessment” OR “Quality Assurance” OR Indicator* OR Metric* OR Indicador*) AND (“Simulation Training” OR “Simulation-based Training” OR “Realistic Simulation” OR “Interactive Training” OR “Interactive Learning” OR “Interactive Simulation” OR “Clinical Simulation” OR “Healthcare Simulation” OR “Clinical Simulation” OR “Simulation Training” OR “Realistic Simulation”)
**LILACS**	(“Quality Indicators” OR “Health Metrics” OR “Quality Metrics” OR “Quality Assessment” OR “Quality Assurance” OR Indicator* OR Metric* OR Indicador*) AND (“Simulation Training” OR “Simulation-based Training” OR “Realistic Simulation” OR “Interactive Training” OR “Interactive Learning” OR “Interactive Simulation” OR “Clinical Simulation” OR “Healthcare Simulation” OR “Clinical Simulation” OR “Simulated Training” OR “Realistic Simulation” OR “Simulated Learning” OR “Simulación Clínica” OR “Entrenamiento Simulado” OR “Simulación Realista”)
**Google Scholar**	(“Quality Indicators” OR “Quality Metrics” OR “Quality Assurance”) AND (“Simulation Training” OR “Clinical Simulation” OR “Healthcare Simulation”)

### Study selection

For study selection, all bibliographic references identified through database and data source searches were exported to Rayyan^®^, where duplicates were removed. Primary studies addressing the management of clinical simulation centers and laboratories, as well as their indicators, were included.

Initially, two reviewers, working independently and anonymously, read the titles and abstracts to verify study eligibility based on the defined inclusion criteria, ensuring that the studies met the study proposal.

In the second phase, the reviewers read the selected articles in full to confirm the inclusion criteria. Disagreements were resolved by consensus or, when necessary, with a third researcher present for evaluation.

### Data extraction

The research team conducted data extraction using a data collection tool aligned with the PCC mnemonic and the proposed review question. It is important to note that this tool is provided in the JBI manual and can be adapted according to the needs of the study authors^9^ ^,^ ^11^.

Based on the PCC mnemonic and with the aim of answering the review question, a data extraction tool was developed consisting of a structured form on the Google Forms^©^ platform to identify and describe the following items: title, objective, year of publication, type of study, country, research object, research question, and quality indicators that could be applied to the management of clinical simulation centers/laboratories, separating them according to the dimensions of structure, processes, and results.

### Separation, summarization, and reporting of results

Data were collected from selected studies, highlighting the relationship between these studies and quality indicators for the management of simulation centers/laboratories. Data extraction allowed us to map, interpret, and analyze the extent, nature, and distribution of the studies included in the review. Next, thematic grouping was performed and categorized according to the classic triad of Structure, Process, and Outcome recommended by Donabedian^7^.

For the proposed study, structure refers to best-practice standards in simulation: pre-briefing (preparation and briefing), operations, professional integrity, simulation design, professional development, and facilitation. In the process, the following are analyzed: simulation design; pre-briefing (preparation and briefing); debriefing; facilitation; professional integrity; continued interprofessional simulation; and learning and performance assessment. The outcome relates to learning and performance assessment standards, the debriefing process, results and objectives, and professional development standards.

A statistical description of the data was also performed in Google Sheets^©^ with the aim of providing an overview of the materials. The entire data collection and study selection procedure was summarized and presented in the results of this review using the PRISMA-ScR flow diagram^10^.

## Results

**Figure 2:**
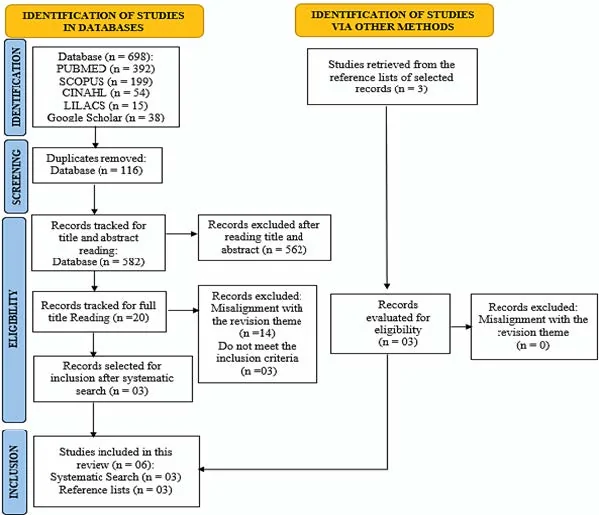
PRISMA-ScR flowchart for study identification. Niterói, RJ, Brazil, 2025

A total of 698 studies were identified from the consultation of relevant information sources and repositories. After eliminating duplicate records, 582 studies remained for subsequent analysis. These studies were evaluated by reading their titles and abstracts, with the aim of excluding those that did not fit the investigated theme. This screening resulted in the selection of 20 studies for full-text evaluation. After reading these materials in full, 17 studies were excluded: 14 lacked alignments with the review topic, and 3 did not meet the predefined inclusion criteria (Figure 2).

After selecting the articles identified in the databases and repositories, the sample for this review comprised six studies (n = 06), published between 2013 and 2024. Of this final sample, three studies (n=03; 50%) were identified through systematic research, and three (n=03; 50%) through the reference lists. Regarding format, five studies (n = 05; 83.33%) were scientific articles and one (n = 01; 16.67%) was a master’s thesis. The limited approach to simulation management, particularly with respect to quality indicators for clinical simulation centers/laboratories, was evident in the small number of studies identified, even after applying a broad time frame.

Figure 3 shows the most relevant data from each article according to the author, year/country/language, type and objective of the study. 

Regarding the study authors’ training, four publications were produced by nurses and two by dental surgeons. All studies constitute primary research.

The only information resources that returned titles for review and analysis were PubMed (three articles) and Google Scholar (two articles and one master’s thesis). The quality indicators from these studies were organized and aligned with the best-practice standards of the International Nursing Association for Clinical Simulation and Learning (INACSL)^18^ (Figure 4). The indicators were then categorized according to Donabedian’s theoretical model of health quality assessment, in the dimensions of structure, process, and outcome, widely used in the analysis of health service quality, which allowed for a systematic approach to the identified indicators.

**Figure 3:** Description of studies included in the scoping review according to the author, year/country/language, type and objective of the study. Niterói, RJ, Brazil, 2025

N	Author	Year/Country/Language	Type of study	Objective
**1**	Santos, 2024^12^	2024 Brazil Portuguese	Qualitative study	To analyze the best practices mentioned by nurse managers in clinical simulation centers/laboratories.
**2**	Montanet, et al., 2023^13^	2023 Spain English	Methodological study	To present to the scientific community the methodology behind a new numerical and digital tool for objectively measuring realism in clinical simulation.
**3**	Montanet, et al., 2020^14^	2020 Spain Spanish	Methodological theory and concepts	To validate universal forms to measure simulation fidelity.
**4**	Yuan, 2020^15^	2020 China English	Descriptive study	To develop Standard Simulated Patient (SSP) training content and assessment indicators and explore their validity and reliability.
**5**	Kable, et al., 2017^16^	2017 Australia English	Observational study	To evaluate a series of simulation sessions using an observation schedule developed from evidence-based quality indicators and compare differences in achieving quality outcomes across participating study sites.
**6**	Arthur, et al., 2013^17^	2013 Australia English	Validation study	To develop a set of quality indicators that would be internationally applicable and could be used to guide the development, implementation, and evaluation of simulation experiences in undergraduate nursing curricula.

**Figure 4:** Clinical simulation quality indicators according to Donabedian’s triad of structure, process, and outcomes^7^ and INACSL’s best practice standards in simulation^18^. Niterói, RJ, Brazil, 2025

Structure indicators	INACSL Standard
Assessment of the facilitation team’s preparation^12^	Pre-briefing^19^
Simulation resource availability rate^12^	Operations^20^
Rate of damaged simulation equipment^12^	
Simulation environment security assessment^12^	Professional Integrity^21^
Diversity and fidelity index of clinical simulation scenarios^12^	Simulation Design^22^
Technology aligned with objectives^16^ ^-^ ^17^	
Environmental loyalty aligned with objectives^16^ ^-^ ^17^	
Realistic equipment and medical record^s(16-17^)	
Physical Characterization of the Simulated Participant^13^ ^-^ ^14^	
Scenographic Realism: Fixed Furniture^13^ ^-^ ^14^	
Scenographic Realism: Portable Non-Electronic Elements^13^ ^-^ ^14^	
Scenographic Realism: Portable Electronic Elements^13^ ^-^ ^14^	
Scenographic Realism: Sound Elements^13^ ^-^ ^14^	
Scenographic Realism: Smells^13^ ^-^ ^14^	
Scenographic Realism: Consumable Sanitary Materials^13^ ^-^ ^14^	
Scenographic Realism: Non-Reusable Sanitary Materials^13^ ^-^ ^14^	
Scenographic Realism: Lighting^13^ ^-^ ^14^	
Realism of the Humanoid Simulator: General Physical Characteristics^13^ ^-^ ^14^	
Realism of the Humanoid Simulator: Clothing^13^ ^-^ ^14^	
Realism of the Humanoid Simulator: Vital Signs and Visible Effects^13^ ^-^ ^14^	
Realism of the Humanoid Simulator: Sound Effects^13^ ^-^ ^14^	
Realism of the Humanoid Simulator: Touch^13^ ^-^ ^14^	
Realism of the Humanoid Simulator: Voice^13^ ^-^ ^14^	
Realism of the Humanoid Simulator: *Moulage* and Makeup^13^ ^-^ ^14^	
Familiarity with curriculum and technology^16^ ^-^ ^17^	
Simulation-trained team^16^ ^-^ ^17^	Professional Development^23^
Facilitators with clinical and pedagogical knowledge^16^ ^-^ ^17^	Facilitation^24^
Alignment with curriculum and course objectives^16^ ^-^ ^17^	Simulation Design^22^
Integrated simulation curriculum matrix^16^ ^-^ ^17^	
Scaling of experiences with progressive support^16^ ^-^ ^17^	
Integration of simulation across all clinical disciplines^16^ ^-^ ^17^	
Learning objectives guide simulation design^16^ ^-^ ^17^	
Knowledge of the disease: symptoms, signs, treatment^15^	
Consistency in each performance^15^	
Development of the script’s plot^15^	
Patient characteristics^15^	
Relevant physical examinations, laboratory tests, and imaging tests^15^	
Realism of Simulated Participant Interpretation: Conceptual Characterization^13^ ^-^ ^14^	
Realism of Simulated Participant Interpretation: Emotional Characterization^13^ ^-^ ^14^	
Realism of Simulated Participant Interpretation: Improvisation^13^ ^-^ ^14^	
Realism of Simulated Participant Interpretation: Relationship with the Student^13^ ^-^ ^14^	
Realism of the Humanoid Simulator: Interaction with the Student^13^ ^-^ ^14^	
Structured guidance for students^16^ ^-^ ^17^	Pre-briefing^19^
Familiarization with mannequins and equipment^16^ ^-^ ^17^	
Briefing on the structure of the session^16^	
Clarity of objectives to students^16^	
Preparatory materials available^16^ ^-^ ^17^	
Prior training in psychomotor skills^16^	
Assessment of readiness for simulation^12^	
Immediate structured debriefing^16^ ^-^ ^17^	The Debriefing Process^25^
Reflection and self-assessment^16^	
Discussion of non-technical skills^16^	
Feedback on strengths^16^	
Feedback on weaknesses^16^	
Combined feedback^16^	
Emotional support after unsatisfactory performance^16^	
Effectiveness rate of facilitation^12^	Facilitation^24^
Each performance is consistent^15^	
Performance is consistent with the plot of the play^15^	
Dealing with unforeseen events^15^	
Memory: medical history and health concerns^15^	
Demonstration of symptoms^15^	
Demonstration of signs^15^	
Demonstration of emotional responses^15^	
Clarity and fluency of expression^15^	
Imitation of tone/intonation, expressions, and postures^15^	
Realistic psychological reactions^15^	
Punctuality and dedication^15^	Professional Integrity^21^
Professional integrity in clinical simulation^12^	
Participation in interprofessional simulations^12^	Interprofessional Simulation^26^
Assessment content^15^	Learning and Performance^27^
Scoring criteria for each indicator^15^	
Improvement in clinical performance after clinical simulation^12^	
NPS* index of student/professional satisfaction with clinical simulation^12^	
Effectiveness of reflection and learning^12^	
Realism Achieved (Objective)^13^ ^-^ ^14^	
Realism Perceived (Subjective)^13^ ^-^ ^14^	
Accuracy of assessment as an evaluator^15^	
Attention to student performance^15^	
Average debriefing time in clinical simulation^12^	The Debriefing Process^25^
Debriefing focused on reflection and soft skills^17^	
Improvement in clinical competence^12^	Results and objectives^28^
Effectiveness in achieving learning objectives in clinical simulations^12^	
Average number of training sessions conducted by simulation professionals for clinical simulation training^12^	Professional development^23^

*Net Promoter Score (NPS) Index

### Structure indicators

In this category, 27 indicators were mapped, primarily based on standards of operations, professional integrity, and simulation design^20^ ^-^ ^22^. The “Simulation Design” standard was the most significant, focusing on metrics related to technology, environmental fidelity, and scenographic realism^12^ ^-^ ^14^ ^,^ ^16^ ^-^ ^17^. It should be noted that most of these indicators aim to ensure that the physical and technological infrastructure aligns with the pedagogical objectives, with an emphasis on preparing the environment and the facilitation team.

### Process indicators

This category comprises 45 indicators, subdivided into the following best-practice standards: simulation design; pre-briefing (preparation and briefing); the debriefing process; facilitation; professional integrity; ongoing interprofessional simulation; and learning and performance assessment^19^ ^,^ ^21^ ^-^ ^22^ ^,^ ^24^ ^-^ ^27^.

The analysis reveals a strong focus on aspects of pedagogical practice, such as curriculum alignment, debriefing, and simulated participant performance^13^ ^-^ ^17^. It was observed that process indicators prioritize interaction between the facilitator and the student, the consistency of simulations, and the assessment of student readiness, reflecting a concern for the integrity of the learning experience.

### Outcome indicators

Twelve indicators were identified in this dimension, with a predominant focus on the learning and performance assessment standard^27^. The most relevant points include measuring improvement in clinical performance after simulation, the user satisfaction index (NPS), and the effectiveness of reflection achieved during debriefing^12^. It should be noted that the results primarily focus on learning outcomes and the simulation team’s continuous training.

## Discussion

This scoping review aimed at mapping quality indicators for the management of clinical simulation centers/laboratories in the literature. The analysis of the six included studies identified a diverse set of indicators, which were categorized according to Donabedian’s classic triad-Structure, Process, and Outcome^7^-and correlated with INACSL best practice standards^18^.

When analyzing the studies included in this review, it is noteworthy that some publications exhibit methodological and conceptual continuity. The research demonstrates its applied nature by using previously defined indicators^16^ ^-^ ^17^ and by deepening and operationalizing the proposed concepts, indicating a trajectory of theoretical maturation regarding fidelity in clinical simulation^13^ ^-^ ^14^.

The comparison of the indicators mapped in this study highlights a substantial gap in the management processes of clinical simulation centers/laboratories. Although elements such as fidelity, realism, pedagogical design, and physical structure of simulated environments are important and widely covered in the selected studies^13^ ^-^ ^17^, it is observed that most of the studies prioritize aspects related to the pedagogical practice of simulation, failing to adequately address the administrative and operational components that support the functioning of clinical simulation centers/laboratories.

Thus, it became evident that, despite the large number of identified indicators, they do not constitute a set that can be directly applied to the full evaluation of the management processes of clinical simulation centers/laboratories, the subject of this study. It is believed that issues such as planning, resource organization, staff training, supply management, equipment maintenance, goal setting, and continuous monitoring are still addressed in an incipient manner in scientific articles. This absence may compromise the ability to assess the quality of management processes, limiting the use of indicators as a strategic management tool.

A study developed and validated, with the help of experts, several quality indicators listed in four domains, referred to respectively as “pedagogical principles”, “student preparation”, “fidelity” and “debriefing”^16^. These indicators are suitable for measuring the quality of the pedagogical aspect and the implementation of the methodology in accordance with the recommendations addressed by the INACSL standards^18^. However, it is understood that they do not provide an objective assessment of the management of clinical simulation centers/laboratories.

Management indicators are not only required for measuring performance but also serve as a strategic tool for maintaining the simulation program. In addition, accreditation processes for simulation programs have increasingly required the presentation of consolidated metrics attesting to the effectiveness of pedagogical and managerial practices^29^.

The accreditation process of the Society for Simulation in Healthcare (SSH) requires clinical simulation centers/laboratories to demonstrate excellence in areas such as governance, financial sustainability, human resources, and facility management^30^.

On the other hand, a study presented indicators in the areas of “student assessment as a simulated patient” and “assessment as an evaluator”, considering metrics such as “Consistency in each performance”, “Development of the script plot”, “Imitation of tone/intonation, expressions, and postures”, and “Clarity and fluency of expression”^15^. Although these indicators do not specifically address the management processes of clinical simulation centers/laboratories, they can be considered by managers as Key Performance Indicators (KPIs), depending on institutional objectives and the stage of development of the simulation program.

A KPI in healthcare is an objective metric used to monitor, evaluate, improve, control, and promote changes in care processes, with the purpose of ensuring the effectiveness, quality, and efficiency of services, as well as increasing patient and healthcare professional satisfaction^31^. It is understood that KPIs in clinical simulation programs should assist managers in analyzing training data and organizing the activities of the clinical simulation center/laboratory.

Following this line of reasoning, a set of indicators focused on the planning and structuring of simulation practices was identified, organized into domains such as “conceptual fidelity”, “physical fidelity”, “emotional fidelity”, “simulated participant”, “scenography”, and “simulator”. Among the various indicators highlighted by the authors, those related to scenographic realism (such as fixed furniture, sound effects, odors, lighting), the realism of the humanoid simulator (physical characteristics, clothing, touch, voice, *moulage*), the interpretation of the simulated participant (covering conceptual and emotional characterization, improvisation, and interaction with the student), as well as aspects of achieved realism and perceived realism^13^ ^-^ ^14^.

Although such metrics are fundamental for assessing realism, improving the effectiveness of simulation-based teaching, and verifying the achievement of proposed educational objectives, they do not directly address the management processes of clinical simulation centers/laboratories. Even so, they can offer relevant insights that allow for extrapolations or reflections applicable to the management of these environments.

In this context, researchers emphasize the role of clinical simulation centers/laboratories as structures that link the university, the health system, and public management. The authors reinforce the relevance of integration among management, teaching, and service, as well as the need to evaluate, based on evidence, the impact of simulation on professional training and the quality of health care^32^.

Thus, indicators that do not directly address the managerial aspects of clinical simulation centers/laboratories can still support management strategies in these environments. The study also proposes the following question: “which indicators can help to investigate the effects of simulation on training and services?”, thus highlighting a gap in the literature that deserves to be explored^32^.

This finding seems to point to a dichotomy between teaching-learning and managerial indicators. Although they are complementary, the processes of planning, operations, sustainability, logistics, staff training, equipment maintenance, and analysis of institutional outcomes require their own indicators that extend beyond the evaluation of student performance.

Some studies have proposed an approach more aligned with the scope of this review, organizing indicators into the domains of “infrastructure”, “technological resources”, “pedagogical principles”, and “preparation of those involved”^16^ ^-^ ^17^. Based on this structure, it is understood that indicators such as “technology aligned with objectives”, “Realistic equipment and medical records”, “Integrated simulation curriculum matrix”, “Available preparatory materials”, as well as “Learning objectives guide simulation design”^16^ ^-^ ^17^, can be considered, albeit indirectly, as components of management processes in clinical simulation centers/laboratories, contributing to the qualification of management and the alignment between pedagogical practices and institutional resources.

In light of this, the Best Practice Standards in Health Simulation establish specific guidelines for the operation of clinical simulation centers/laboratories. It is believed that these guidelines can serve as a reference for the formulation of quality indicators, covering both the pedagogical dimension and aspects related to the management of these spaces^20^.

The “Operations” standard established by INACSL emphasizes that “all simulation-based education programs require systems and infrastructure to support and maintain operations”^20^. This guideline underscores the importance of well-defined organizational practices to ensure the sustainability and quality of activities conducted in clinical simulation centers/laboratories.

In this regard, one study proposed indicators directly focused on the management of clinical simulation centers/laboratories, organized into the domains of “structure”, “process”, and “results”. Examples include the “simulation resource availability rate”, the “rate of damaged simulation equipment”, the “assessment of the safety of the simulation environment”, “improvement in clinical performance after clinical simulation”, and “student/professional satisfaction with clinical simulation”^12^. However, these indicators still require further methodological refinement and validation by experts in the field.

It is believed that the development and validation of consistent and applicable indicators can contribute to the management of clinical simulation centers/laboratories, thereby promoting organizational control, continuous improvement, institutional transparency, and alignment with international benchmarks, such as those proposed by INACSL^18^. However, effective implementation of these indicators may be hindered by the particularities and limitations of each institution.

In this context, it should also be noted that developing a set of policies and procedures for the operation of clinical simulation centers/laboratories is a challenge that requires time and resource investment. Among the main obstacles are: the lack of efficient information systems for data collection and analysis; institutional resistance to adopting changes and implementing new monitoring mechanisms; the absence of consolidated comparative parameters (benchmarks) for performance evaluation; difficulties in measuring the impact of management practices on educational outcomes; and disparities in the resources available between institutions^29^.

Overcoming these challenges requires collaborative and flexible strategies that account for the specificities of each institutional context in the articulation among management, teaching, and service^29^ ^,^ ^31^. In this sense, it is understood that, after defining management processes-preferably based on national and international benchmarks-it is essential to conduct studies to validate specific indicators for the management of clinical simulation centers/laboratories.

Among the six studies included in this research sample, only one specifically addressed the development of indicators for the management of clinical simulation centers/laboratories^12^. However, these indicators lack validation by specialists.

Among the study’s limitations, we highlight the nascent state of the scientific literature on quality indicators in clinical simulation, particularly those related to management processes in clinical simulation centers/laboratories. In addition, the articles analyzed present a diversity of contexts, objectives, and methodological formats, which made it difficult to categorize and directly compare the indicators.

As implications for advancing scientific knowledge in health and nursing, the study offers an overview for clinical simulation centers and laboratories/laboratories and for nursing managers, on how to structure evaluation criteria for simulation programs based on indicators, thereby promoting greater objectivity and standardization. In addition, fostering discussion on the integration of pedagogical planning and simulation management can support institutional criteria, reinforcing a culture of quality and safety in health education.

## Conclusion

The study mapped the scientific literature on management process indicators in clinical simulation centers/laboratories, guided by the research question: What quality indicators are necessary for the management of clinical simulation centers/laboratories?

The findings support clinical simulation centers/laboratories in structuring evaluation criteria based on indicators, thereby promoting greater efficiency and alignment between management and educational practices and enhancing the communication of results. Nevertheless, a significant gap was evident in the definition and validation of indicators specifically designed to manage these environments. It was found that, although the topic is fundamental to the sustainability and effectiveness of these training spaces, most publications focus on pedagogical aspects of simulation, such as realism, instructional design, and student assessment, paying little attention to administrative and operational dimensions.

Given this scenario, there is a clear need to encourage methodological and applied research to develop validated, internationally aligned indicators for quality and accreditation. It is believed that developing a system of indicators focused on management processes can promote transparency, increase efficiency, and strengthen the strategic alignment between pedagogical practices and the institutional management of clinical simulation centers/laboratories.

It is therefore recommended that future research advance in the proposal and validation of management process indicators in these environments, considering their particularities and the challenges inherent to their operation, with the aim of continuously improving teaching and learning spaces in health.

It is also suggested that quantitative studies be conducted to evaluate the quality of clinical simulation, with the results informing the optimization of resources, decision-making, and management planning in line with the real needs of the health sector.

## Data Availability

All data generated or analysed during this study are included in this published article.

## References

[1] Diaz-Navarro C, Armstrong R, Charnetski M, Freeman KJ, Koh SB, Reedy G (2024). Global consensus statement on simulation-based practice in healthcare. Adv Simul.

[2] Oman SP, Magdi Y, Simon LV (2023). Past, Present, and Future of Simulation in Internal Medicine. StatPearls [Internet].

[3] Ayaz O, Ismail FW (2022). Healthcare simulation: a key to the future of medical education - a review. Adv Med Educ Pract.

[4] Flores-Cohaila JA, Ñaña-Cordova AM, Rios-Garcia W, Benavente-Chalco XC, Torres-Zegarra BC, Bustamante-Ordoñez MA (2024). Low-cost simulation in health professions education: a bibliometric analysis and literature review of 20 years of research. Educ Med.

[5] Xu X, Yao J, Bohnert J, Yamada N, Lee HC (2024). Implementation of a multi-site neonatal simulation improvement program: a cost analysis. BMC Health Serv Res.

[6] Mehrolhassani MH, Behzadi A, Asadipour E (2025). Key performance indicators in emergency department simulation: a scoping review. Scand J Trauma Resusc Emerg Med.

[7] Donabedian A, Donabedian A (1980). Explorations in quality assessment and monitoring. Vol. 1, The definition of quality and approaches to its assessment.

[8] Donabedian A (1990). The seven pillars of quality. Arch Pathol Lab Med.

[9] Peters MDJ, Godfrey CM, McInerney P, Munn Z, Tricco AC, Khalil H, Aromataris E, Munn Z (2020). JBI Manual for Evidence Synthesis [Internet].

[10] Tricco AC, Lillie E, Zarin W, O’Brien KK, Colquhoun H, Levac D (2018). PRISMA extension for scoping reviews (PRISMA-ScR): checklist and explanation. Ann Intern Med.

[11] Pollock D, Peters MDJ, Khalil H, McInerney P, Alexander L, Tricco AC (2023). Recommendations for the extraction, analysis, and presentation of results in scoping reviews. JBI Evid Synth.

[12] Santos ISN (2024). Gestão de laboratórios de simulação clínica em saúde: análise de boas práticas [thesis].

[13] Coro-Montanet G, Pardo Monedero MJ, Sánchez Ituarte J, Rocha HWP, Gomar Sancho C (2023). Numerical assessment tool to measure realism in clinical simulation. Int J Environ Res Public Health.

[14] Coro-Montanet G, Bartolomé-Villar B, García-Hoyos F, Pardo Monedero MJ, Sánchez Ituarte J, Gomar Sancho C (2020). Indicadores para medir fidelidad en escenarios simulados. FEM Rev Fund Educ Med.

[15] Yuan HB (2021). Development of student simulated patient training and evaluation indicators in a high-fidelity nursing simulation: a Delphi consensus study. Front Nurs.

[16] Kable AK, Levett-Jones TL, Arthur C, Reid-Searl K, Humphreys M, Morris S (2017). A cross-national study to objectively evaluate the quality of diverse simulation approaches for undergraduate nursing students. Nurse Educ Pract.

[17] Arthur C, Levett-Jones T, Kable AK (2013). Quality indicators for the design and implementation of simulation experiences: a Delphi study. Nurse Educ Today.

[18] INACSL Standards Committee (2021). Healthcare Simulation Standards of Best Practice™. Clin Simul Nurs.

[19] INACSL Standards Committee (2021). Healthcare Simulation Standards of Best Practice™ Prebriefing: Preparation and Briefing. Clin Simul Nurs.

[20] INACSL Standards Committee (2021). Healthcare Simulation Standards of Best Practice™ Operations. Clin Simul Nurs.

[21] Bowler F, Klein M, Wilford A, INACSL Standards Committee (2021). Healthcare Simulation Standards of Best Practice™ Professional Integrity. Clin Simul Nurs.

[22] Watts PI, McDermott DS, Alinier G, Charnetski M, Ludlow J, INACSL Standards Committee (2021). Healthcare Simulation Standards of Best Practice™ Simulation Design. Clin Simul Nurs.

[23] Hallmark B, Brown M, Peterson DT, Fey M, Morse C, INACSL Standards Committee (2021). Healthcare Simulation Standards of Best Practice™ Professional Development. Clin Simul Nurs.

[24] Persico L, Belle A, DiGregorio H, Wilson-Keates B, Shelton C, INACSL Standards Committee (2021). Healthcare Simulation Standards of Best Practice™ Facilitation. Clin Simul Nurs.

[25] Decker S, Alinier G, Crawford SB, Gordon RM, Jenkins D, INACSL Standards Committee (2021). Healthcare Simulation Standards of Best Practice™ The Debriefing Process. Clin Simul Nurs.

[26] Rossler K, Molloy MA, Pastva AM, Brown KM, Xavier N, INACSL Standards Committee (2021). Healthcare Simulation Standards of Best Practice™ Simulation-Enhanced Interprofessional Education. Clin Simul Nurs.

[27] McMahon E, Jimenez FA, Lawrence K, Victor J, INACSL Standards Committee (2021). Healthcare Simulation Standards of Best Practice™ Evaluation of Learning and Performance. Clin Simul Nurs.

[28] Miller C, Deckers C, Jones M, Wells-Beede E, McGee E, INACSL Standards Committee (2021). Healthcare Simulation Standards of Best Practice™ Outcomes and Objectives. Clin Simul Nurs.

[29] Khan M, Sasso RA (2024). Obtaining Medical Simulation Center Accreditation. StatPearls [Internet].

[30] Society for Simulation in Healthcare Accreditation, Committee for Accreditation of Healthcare Programs (2021). Core Accreditation Standards Companion Document.

[31] Varela T, Zamorano P, Muñoz P, Rain C, Irazoquí E, Sapag JC (2023). Evaluation of the implementation progress through key performance indicators in a new multimorbidity patient-centered care model in Chile. BMC Health Serv Res.

[32] Mazzo A, Oliveira-Costa RR, Manzoni-Lourençone LF, Santos-Almeida RG, Henrique-Sanches BC (2022). Skills and simulation laboratory: current and future perspectives. Rev Latinoam Simul Clín.

