# Supplementary material for: Quality indicators for the management of clinical simulation centers and laboratories: a scoping review
Source: Rev Lat Am Enfermagem. 2026 Aug 11;34:e4906. doi: 10.1590/1518-8345.8215.4906 (PMC13505610; doi:10.1590/1518-8345.8215.4906)
Supplement: Translation

# Translation: Indicadores de qualidade para gestão de centros e laboratórios de simulação clínica: revisão de escopo

**DOI:** 10.1590/1518-8345.8215.4907

**Authors:** Isaac Sebastião Nunes Santos, Cláudio José de Souza, Rodrigo Leite Hipolito, Márcia Farias de Oliveira, Carolina Baptista Ribeiro, André da Silva Brites

**Affiliations:** 1 Universidade Federal Fluminense, Escola de Enfermagem Aurora de Afonso Costa, Niterói, RJ, Brasil.; 2 Hospital Universitário Antônio Pedro, Centro de Terapia Intensiva, Niterói, RJ, Brasil.

**Keywords:** Treinamento por simulação, Indicadores de Gestão, Indicadores de Qualidade em Assistência à Saúde, Garantia da Qualidade dos Cuidados de Saúde, Organização e Administração, Gestão em Saúde

## Abstract

**Objetivo:**

mapear na literatura os indicadores de qualidade para gestão de centros e laboratórios de simulação clínica.

**Método:**

revisão de escopo conduzida conforme diretrizes do JBI e da extensão *Preferred Reporting Items for Systematic Reviews and Meta-Analyses for Scoping Reviews*. Realizaram-se buscas sobre indicadores de qualidade para gestão de centros/laboratórios de simulação clínica, publicados desde 1990, em quatro fontes de informação e no *Google Scholar*, sem restrição de método, idioma ou origem. Os dados foram extraídos após agrupamento temático e categorizados segundo a tríade Estrutura, Processo e Resultado, preconizada por Donabedian, correlacionando-os com os padrões de melhores práticas da *International Nursing Association for Clinical Simulation and Learning*.

**Resultados:**

dos seis estudos selecionados, entre 2013 e 2024, foram mapeados oitenta e quatro indicadores de qualidade. Apesar do elevado número de indicadores, estes não são plenamente aplicáveis à avaliação dos processos gerenciais dos centros/laboratórios de simulação clínica. Planejamento, organização de recursos, capacitação da equipe, gerenciamento de insumos, manutenção de equipamentos e monitoramento contínuo ainda são incipientes na literatura.

**Conclusão:**

recomenda-se em investigações futuras a validação de indicadores de processos gerenciais dos centros/laboratórios de simulação clínica, considerando suas particularidades e desafios operacionais, objetivando a qualificação contínua dos espaços de ensino-aprendizagem em saúde.

## Destaques

**(1)** Identificados 84 indicadores de qualidade para gestão de centros de simulação clínica. **(2)** Os indicadores de avaliação ainda são pouco aplicáveis aos processos gerenciais **(3)** Predominio de indicadores pedagógicos sobre gerenciais. **(4)** Subsídios para estruturação de indicadores específicos para avaliação gerencial. **(5)** Necessidade de validação de indicadores gerenciais em centros de simulação clínica.

## Introdução

A simulação clínica se consolidou como uma ferramenta para o ensino e a capacitação de alunos/profissionais da saúde. Trata-se de uma técnica que cria uma situação ou ambiente simulado para permitir que os profissionais de saúde experimentem a representação de um evento real, possibilitando a prática, o aprendizado, a avaliação, teste ou compreensão de sistemas ou ações humanas. Além dos benefícios diretos para a educação e o treinamento de alunos/profissionais de saúde, a simulação clínica também favorece a otimização dos sistemas de saúde, melhorando a assistência prestada e contribuindo para a segurança do paciente^1^. 

O uso da simulação na educação em saúde teve início no século XVIII, com manequins anatômicos voltados ao ensino de obstetrícia na França; obteve, porém, maior impulso a partir da década de 1950, com o desenvolvimento de simuladores eletrônicos de baixa e alta fidelidade que permitem reproduzir funções fisiológicas, responder aos medicamentos e ensinar o manejo básico das vias aéreas^2^ ^-^ ^3^. Nas décadas seguintes, o avanço tecnológico resultou em simuladores cada vez mais sofisticados, transformando a formação em saúde e estimulando a criação de centros e laboratórios de simulação; contudo, o elevado custo de equipamentos e *softwares* especializados permanece um desafio para instituições com recursos limitados^4^.

Diante desse cenário, é necessário considerar que a implementação de um programa de simulação requer um investimento significativo de recursos materiais e humanos, além de tempo. Isso deve ser reconhecido e cuidadosamente considerado antes do início de um programa de simulação por meio de uma análise de custo-efetividade baseada nos possíveis benefícios na prática clínica, além dos desfechos dos pacientes. Assim, a eficácia por si só não justifica os altos investimentos, sendo imprescindível analisar também a eficiência, ou seja, o quanto de valor é gerado em relação ao custo empregado^5^.

Diante da expansão dos centros/laboratórios de simulação clínica nas instituições de ensino e nos serviços de saúde, torna-se imprescindível que esses ambientes sejam gerenciados de forma eficaz, de modo a assegurar sua relevância no processo educacional e seu impacto positivo na formação profissional. Para tanto, é fundamental que a gestão desses espaços incorpore, entre outras estratégias, o desenvolvimento e a utilização de indicadores de qualidade, que permitam monitorar e avaliar seu funcionamento, desempenho e contribuição para o aprimoramento do ensino^6^.

O uso de indicadores de qualidade tem se consolidado como uma prática recorrente na administração dos serviços de saúde, uma vez que possibilita tanto a avaliação dos resultados quanto a melhoria contínua das atividades pedagógicas e operacionais^7^. No âmbito dos centros/laboratórios de simulação, é importante que esses indicadores abranjam elementos relacionados à estrutura, aos processos e aos resultados, contemplando aspectos como a infraestrutura disponível, a formação dos instrutores, as metodologias adotadas e os resultados observados na aprendizagem dos alunos/profissionais de saúde.

Indicadores de qualidade, sejam eles qualitativos, sejam quantitativos, são imprescindíveis no apoio à tomada de decisões estratégicas. A sua seleção e utilização devem ser cuidadosamente planejadas, ao considerar as especificidades dos centros e laboratórios de simulação clínica, os requisitos relacionados à acreditação e os objetivos estratégicos previamente definidos^8^.

Acredita-se que a crescente implementação de centros/laboratórios de simulação clínica demanda uma gestão eficiente e baseada em evidências. No entanto, a literatura carece de estudos que abordem indicadores específicos relacionados aos processos gerenciais desses ambientes. A carência desses parâmetros compromete a avaliação da eficiência e a melhoria contínua. Este estudo justifica-se pela necessidade de mapear indicadores de qualidade aplicáveis à gestão de centros/laboratórios de simulação clínica, contribuindo para sua sustentabilidade, alinhamento estratégico e qualificação das práticas educacionais. Diante deste contexto, o objetivo deste estudo foi mapear na literatura os indicadores de qualidade para gestão de centros e laboratórios de simulação clínica.

## Método

### Tipo de revisão 

Esta revisão de escopo segue as diretrizes e recomendações do manual do JBI e as orientações do *Preferred Reporting Items for Systematic Reviews and Meta-Analyses Extension for Scoping Reviews* (PRISMA-ScR)^9^ ^-^ ^10^.

Este formato de revisão permite mapear e reunir uma grande diversidade de materiais relacionados a um tema específico, oferecendo uma compreensão ampla do assunto. Também possibilita a consideração de estudos com variados níveis de evidência e diferentes métodos, incluindo fontes de informação e literatura cinzenta. Contudo, não se destina a realizar avaliação crítica, nem análise do risco de viés ou hierarquização dos níveis de evidência^9^.

Para garantir a confiabilidade e a lisura do processo de revisão, o protocolo foi registrado no *Open Science Framework* (https://doi.org/10.17605/OSF.IO/6AGBE).

### Critérios de elegibilidade

Nesta revisão, foram incluídos estudos originais cujos objetos abordaram indicadores de qualidade para a gestão de centros/laboratórios de simulação clínica. Foram considerados os estudos publicados a partir de 1990, considerando que este foi o período de maior desenvolvimento da simulação clínica. Não houve restrição quanto ao tipo de delineamento metodológico, idioma ou ao país de origem dos estudos, sendo incluídas as publicações que atenderam à pergunta de pesquisa e aos critérios de inclusão definidos com base na estratégia mnemônica População - Contexto - Conceito (PCC), aplicada em revisões de escopo. Foram excluídos os estudos sem adesão ao tema, estudos que não atenderam integralmente aos componentes PCC estabelecidos e publicações duplicadas entre as bases de dados.

Conforme as diretrizes do manual do JBI, o mnemônico PCC deve seguir o padrão de referência (População, Conceito e Contexto) relevante para definição do título claro e essa definição precisa aparecer de forma idêntica no objetivo, pergunta de pesquisa e critérios de inclusão. Neste estudo, População = centros/laboratórios de simulação clínica; Conceito = indicadores de qualidade/gestão; Contexto = gestão e monitoramento desses centros. Assim, a pergunta de revisão estabelecida foi: Quais são os indicadores de qualidade necessários para a gestão de centros/laboratórios de simulação clínica?

### Fontes de informação

A busca por artigos foi realizada em fontes de informação de literatura científica, incluindo o *Medical Literature Analysis and Retrieval System Online* (MEDLINE) via PubMed, o *Cumulative Index to Nursing and Allied Health Literature* (CINAHL), a Literatura Latino-americana e do Caribe em Ciências da Saúde (LILACS) e a Scopus. Além disso, o Google *Scholar* foi utilizado para a busca de teses e dissertações (literatura cinzenta), como também a lista de referências.

### Estratégias de busca

As estratégias de busca foram desenvolvidas pelo autor principal, em conjunto com seu orientador e com o suporte de um bibliotecário especializado na construção de estratégias de busca para revisões de escopo. Para garantir a adequação dos termos e fontes, foi realizada uma consulta preliminar em 15 de agosto de 2024.

A estratégia de busca foi conduzida associando descritores e termos livres sinônimos correspondentes aos elementos do acrônimo PCC, com o objetivo de identificar evidências científicas que respondam à questão de pesquisa deste estudo. Para a seleção dos termos, foram consultados os vocabulários controlados da área da saúde, incluindo os Descritores em Ciências da Saúde (DeCS) e o*Medical Subject Headings* (MeSH).

Foram incluídos os estudos publicados a partir de 1990, considerando que este foi o período de maior desenvolvimento da simulação clínica. Não foram aplicados filtros quanto ao idioma ou desenho do estudo, garantindo uma busca ampla e abrangente. Além disso, as estratégias de busca foram elaboradas seguindo as diretrizes do *Peer Review of Electronic Search Strategies* (PRESS), assegurando a qualidade e a rigorosidade do processo.

As estratégias de busca foram desenvolvidas a partir de uma busca preliminar, seguida da análise dos títulos, resumos e palavras-chave dos estudos recuperados, o que possibilitou o refinamento e a seleção dos termos relacionados aos indicadores de qualidade em saúde e ao treinamento baseado em simulação. Para responder à pergunta de revisão, foram utilizadas combinações dos descritores *“Quality Indicators, Health Care”* e *“Quality Assurance, Health Care”*, bem como termos livres e descritores controlados associados à simulação clínica e ao treinamento por simulação. As buscas foram estruturadas com o uso de operadores *booleanos* e adaptadas às especificidades técnicas de cada base de dados, incluindo variações linguísticas quando pertinente, conforme apresentado na Figura 1.

**Figura 1:** Estratégias de busca nas bases de dados pesquisadas. Niterói, RJ, Brasil, 2025

**Estratégias de busca**	
**PubMed**	(Quality Indicators, Health Care[mj] OR Quality Assurance, Health Care[mj] OR Quality Indicator*[tiab] OR Health Metric*[tiab] OR Quality Metric*[tiab] OR Quality Assessment[tiab] OR Quality Assurance[tiab] OR Indicator*[ti] OR Metric*[ti]) AND (Simulation Training[mj] OR Simulation Training[tiab] OR Simulation-based Training[tiab] OR Realistic Simulat*[tiab] OR Interactive Training[tiab] OR Interactive Learning[tiab] OR Interactive Simulat*[tiab] OR Clinical Simulat*[tiab] OR Healthcare Simulat*[tiab] OR Patient Simulat*[tiab])
**Scopus**	TITLE-ABS(“Quality Indicator*” OR “Health Metric*” OR “Quality Metric*” OR “Quality Assessment” OR “Quality Assurance” OR Indicator* OR Metric*) AND TITLE(“Simulation Training” OR “Simulation-based Training” OR “Realistic Simulation” OR “Interactive Training” OR “Interactive Learning” OR “Interactive Simulation” OR “Clinical Simulation” OR “Healthcare Simulation” OR “Patient Simulation”)
**CINAHL**	(“Quality Indicators” OR “Health Metrics” OR “Quality Metrics” OR “Quality Assessment” OR “Quality Assurance” OR Indicator* OR Metric* OR Indicador*) AND (“Simulation Training” OR “Simulation-based Training” OR “Realistic Simulation” OR “Interactive Training” OR “Interactive Learning” OR “Interactive Simulation” OR “Clinical Simulation” OR “Healthcare Simulation” OR “Simulação Clinica” OR “Treinamento de Simulação” OR “Simulação Realística”)
**LILACS**	(“Quality Indicators” OR “Health Metrics” OR “Quality Metrics” OR “Quality Assessment” OR “Quality Assurance” OR Indicator* OR Metric* OR Indicador*) AND (“Simulation Training” OR “Simulation-based Training” OR “Realistic Simulation” OR “Interactive Training” OR “Interactive Learning” OR “Interactive Simulation” OR “Clinical Simulation” OR “Healthcare Simulation” OR “Simulação Clinica” OR “Treinamento Simulado” OR “Simulação Realística” OR “Aprendizado Simulado” OR “Simulación Clínica” OR “Entrenamiento Simulado” OR “Simulación Realista”)
**Google Scholar**	(“Quality Indicators” OR “Quality Metrics” OR “Quality Assurance”) AND (“Simulation Training” OR “Clinical Simulation” OR “Healthcare Simulation”)

### Seleção dos estudos

Para a seleção dos estudos, todas as referências bibliográficas identificadas por meio das estratégias de busca nas bases e fontes de dados foram exportadas para o Rayyan^®^, onde as duplicatas foram eliminadas. Foram incluídos os estudos primários que abordaram a gestão de centros/laboratórios de simulação clínica e seus indicadores.

Inicialmente, dois revisores realizaram, de forma anônima e independente, a leitura dos títulos e resumos para verificar a elegibilidade dos estudos com base nos critérios de inclusão definidos, garantindo que atenderiam à proposta do estudo.

Na segunda fase, os revisores realizaram a leitura completa dos artigos selecionados, para confirmação dos critérios de inclusão. As discordâncias foram resolvidas por consenso ou com a presença de um terceiro pesquisador para avaliação.

### Extração de dados

O processo de extração de dados foi conduzido pela equipe de pesquisadores, utilizando um instrumento de coleta de dados alinhado com o mnemônico PCC e com a pergunta de revisão proposta. É importante destacar que esse instrumento é fornecido pelo manual da JBI e pode ser adaptado conforme as necessidades dos autores do estudo^9^ ^,^ ^11^.

Com base no mnemônico PCC e com a finalidade de responder à pergunta de revisão, foi desenvolvido um instrumento de extração de dados que consiste em um formulário estruturado na plataforma Google *Forms* ^*©*^ para identificar e descrever os seguintes itens: título, objetivo, ano de publicação, tipo de estudo, país, objeto de pesquisa, questão de pesquisa e indicadores de qualidade que pudessem ser aplicados à gestão de centros/laboratórios de simulação clínica, separando-os segundo as dimensões estrutura, processos e resultados.

### Separação, sumarização e relatório dos resultados

Os dados foram coletados a partir de estudos selecionados, destacando a relação desses estudos com os indicadores de qualidade para a gestão de centros/laboratórios de simulação. A extração dos dados permitiu mapear, interpretar e realizar a análise da extensão, natureza e distribuição dos estudos incluídos na revisão. Em seguida, foram realizados o agrupamento temático e categorizados segundo a tríade clássica Estrutura, Processo e Resultado preconizada por Donabedian^7^. 

Para o estudo proposto, a estrutura refere-se aos padrões de melhores práticas em simulação: *pré-briefing* (preparação e *briefing*); operações; integridade profissional; *design* da simulação; desenvolvimento profissional e facilitação. No processo, analisam-se: *design* da simulação; *pré-briefing* (preparação e *briefing*); o processo do *debriefing*; facilitação; integridade profissional; simulação interprofissional continuada e avaliação da aprendizagem e de desempenho. O resultado relaciona-se aos padrões de avaliação da aprendizagem e desempenho, o processo de *debriefing*, resultados e objetivos, além do padrão de desenvolvimento profissional. 

Também foi realizada a descrição estatística dos dados utilizando o Planilhas Google^©^, com o objetivo de fornecer uma visão geral do conjunto de materiais. Todo o procedimento de coleta de dados e seleção dos estudos foi sumarizado e apresentado nos resultados desta revisão, por meio do diagrama de fluxo PRISMA-ScR^10^.

## Resultados

Um total de 698 estudos foram identificados a partir da consulta às fontes de informação e repositórios relevantes. Após a eliminação de registros duplicados, restaram 582 estudos para análise subsequente. Procedeu-se à avaliação desses estudos por meio da leitura de seus títulos e resumos, com o objetivo de excluir aqueles que não se enquadravam na temática investigada. Essa triagem resultou na seleção de 20 estudos para avaliação integral do texto. Concluída a leitura completa, 17 estudos foram excluídos, sendo que 14 por apresentarem falta de alinhamento com o tema da revisão e 3 por não satisfazerem os critérios de inclusão predefinidos (Figura 2).

**Figura 2:** Fluxograma PRISMA-ScR de identificação dos estudos. Niterói, RJ, Brasil, 2025

Após o processo de seleção dos artigos identificados nas bases de dados e repositórios, a amostra desta revisão compreendeu seis estudos (n = 06), publicados no período de 2013 a 2024. Desta amostra final, 3 (n=03; 50%) estudos originaram-se das buscas sistemáticas realizadas, enquanto 3 (n=03; 50%) foram identificados por meio da lista de referências. Em relação ao formato, 5 (n=05; 83,33%) estudos eram artigos científicos e 1 (n=01; 16,67%), dissertação de mestrado. A limitada abordagem da temática da gestão em simulação, particularmente no que concerne aos indicadores de qualidade de centros/laboratórios de simulação clínica, tornou-se evidente diante do reduzido número de trabalhos encontrados, mesmo após a aplicação de um recorte temporal abrangente.

A Figura 3 mostra os dados mais relevantes de cada artigo segundo o autor, ano/pais/idioma, tipo e objetivo do estudo.

Quanto à formação dos autores, observa-se que quatro publicações foram produzidas por enfermeiros e duas por cirurgiões-dentistas. Todos os estudos constituem pesquisas primárias.

Os únicos recursos informacionais que retornaram títulos para revisão e análise foram a PubMed (três artigos) e Google *Scholar* (dois artigos e uma dissertação de mestrado). Os indicadores de qualidade provenientes destes estudos foram organizados e correlacionados com os padrões de melhores práticas da *International Nursing Association for Clinical Simulation and Learning* (INACSL)^18^. Em seguida, os indicadores foram categorizados segundo o modelo teórico de avaliação da qualidade em saúde proposto por Donabedian, nas dimensões estrutura, processo e resultado, amplamente utilizado na análise da qualidade dos serviços de saúde, o que permitiu uma abordagem sistemática dos indicadores identificados (Figura 4).

**Figura 3:** Descrição dos estudos incluídos na revisão de escopo segundo o autor, ano/pais/idioma, tipo e objetivo do estudo. Niterói, RJ, Brasil, 2025

n	Autor	Ano/País/ Idioma	Tipo de estudo	Objetivo
**1**	Santos, 2024^12^	2024 Brasil Português	Estudo qualitativo	Analisar as boas práticas referidas pelos enfermeiros gestores nos centros/laboratórios de simulação clínica.
**2**	Montanet, et al., 2023^13^	2023 Espanha Inglês	Estudo metodológico	Apresentar à comunidade científica a metodologia por trás de uma nova ferramenta numérica e digital para medir objetivamente o realismo em simulação clínica.
**3**	Montanet, et al., 2020^14^	2020 Espanha Espanhol	Teórico-conceitual metodológico	Validar formulários universais para medir a fidelidade da simulação.
**4**	Yuan, 2020^15^	2020 China Inglês	Estudo descritivo	Construir o conteúdo do treinamento de Paciente Simulado Padrão (PSP) e indicadores de avaliação, além de explorar sua validade e confiabilidade.
**5**	Kable, et al., 2017^16^	2017 Austrália Inglês	Estudo observacional	Avaliar uma série de sessões de simulação utilizando um cronograma de observação desenvolvido a partir de indicadores de qualidade baseados em evidências e comparar as diferenças na obtenção de resultados de qualidade nos locais de estudo participantes.
**6**	Arthur, et al., 2013^17^	2013 Austrália Inglês	Estudo de validação	Desenvolver um conjunto de indicadores de qualidade que fossem aplicáveis internacionalmente e que pudessem ser utilizados para orientar o desenvolvimento, a implementação e a avaliação de experiências de simulação em currículos de enfermagem de graduação.

**Figura 4:** Indicadores de qualidade de simulação clínica segundo a tríade estrutura, processo e resultados de Donabedian^7^ e os padrões de melhores práticas em simulação da INACSL^18^. Niterói, RJ, Brasil, 2025

Indicadores de estrutura	Padrão INACSL
Avaliação da preparação da equipe de facilitação^12^	Pré-*briefing* ^19^
Taxa de disponibilidade de recursos de simulação^12^	Operações^20^
Taxa de equipamentos de simulação avariados^12^	
Avaliação da segurança do ambiente de simulação^12^	Integridade Profissional^21^
Índice de diversidade e fidelidade de cenários de simulação clínica^12^	*Design* da Simulação^22^
Tecnologia alinhada aos objetivos^16^ ^-^ ^17^	
Fidelidade ambiental alinhada aos objetivos^16^ ^-^ ^17^	
Equipamentos e prontuários realista^s(16-17^)	
Caracterização Física do Participante Simulado^13^ ^-^ ^14^	
Realismo Cenográfico: Mobiliário Fixo^13^ ^-^ ^14^	
Realismo Cenográfico: Elementos Portáteis Não Eletrônicos^13^ ^-^ ^14^	
Realismo Cenográfico: Elementos Portáteis Eletrônicos^13^ ^-^ ^14^	
Realismo Cenográfico: Elementos Sonoros^13^ ^-^ ^14^	
Realismo Cenográfico: Odores^13^ ^-^ ^14^	
Realismo Cenográfico: Material Sanitário Fungível^13^ ^-^ ^14^	
Realismo Cenográfico: Material Sanitário Não Fungível^13^ ^-^ ^14^	
Realismo Cenográfico: Iluminação^13^ ^-^ ^14^	
Realismo do Simulador Humanoide: Características Físicas Gerais^13^ ^-^ ^14^	
Realismo do Simulador Humanoide: Vestimenta^13^ ^-^ ^14^	
Realismo do Simulador Humanoide: Sinais Vitais e Efeitos Visíveis^13^ ^-^ ^14^	
Realismo do Simulador Humanoide: Efeitos Sonoros^13^ ^-^ ^14^	
Realismo do Simulador Humanoide: Tato^13^ ^-^ ^14^	
Realismo do Simulador Humanoide: Voz^13^ ^-^ ^14^	
Realismo do Simulador Humanoide: *Moulage* e Maquiagem^13^ ^-^ ^14^	
Familiaridade com currículo e tecnologia^16^ ^-^ ^17^	
Equipe treinada em simulação^16^ ^-^ ^17^	Desenvolvimento profissional^23^
Facilitadores com conhecimento clínico e pedagógico^16^ ^-^ ^17^	Facilitação^24^
Alinhamento com currículo e objetivos do curso^16^ ^-^ ^17^	*Design* da Simulação^22^
Matriz curricular da simulação integrada^16^ ^-^ ^17^	
Escalonamento de experiências com suporte progressivo^16^ ^-^ ^17^	
Integração da simulação em todas as disciplinas clínicas^16^ ^-^ ^17^	
Objetivos de aprendizagem guiam o *design* da simulação^16^ ^-^ ^17^	
Conhecimento da doença: sintomas, sinais, tratamento^15^	
Consistência em cada performance^15^	
Desenvolvimento do enredo do roteiro^15^	
Características do paciente^15^	
Exames físicos relevantes, e exames laboratoriais e de imagem^15^	
Realismo da Interpretação do Participante Simulado: Caracterização Conceitual^13^ ^-^ ^14^	
Realismo da Interpretação do Participante Simulado: Caracterização Emocional^13^ ^-^ ^14^	
Realismo da Interpretação do Participante Simulado: Improvisação^13^ ^-^ ^14^	
Realismo da Interpretação do Participante Simulado: Relação com o Aluno^13^ ^-^ ^14^	
Realismo do Simulador Humanoide: Interação com o Aluno^13^ ^-^ ^14^	
Orientação estruturada ao estudante^16^ ^-^ ^17^	Pré*-briefing* ^19^
Familiarização com manequins e equipamentos^16^ ^-^ ^17^	
*Briefing* sobre a estrutura da sessão^16^	
Clareza dos objetivos aos estudantes^16^	
Materiais preparatórios disponíveis^16^ ^-^ ^17^	
Treinamento prévio de habilidades psicomotoras^16^	
Avaliação da prontidão para a simulação^12^	
*Debriefing* estruturado imediato^16^ ^-^ ^17^	O Processo do *Debriefing* ^25^
Reflexão e autoavaliação^16^	
Discussão de habilidades não técnicas^16^	
*Feedback* sobre pontos fortes^16^	
*Feedback* sobre pontos fracos^16^	
*Feedback* combinado^16^	
Suporte emocional após desempenho insatisfatório^16^	
Taxa de efetividade da facilitação^12^	Facilitação^24^
Cada performance é consistente^15^	
A performance é consistente com o enredo da peça^15^	
Lidar com eventos imprevistos^15^	
Memória: histórico médico e preocupações com a saúde^15^	
Demonstração de sintomas^15^	
Demonstração de sinais^15^	
Demonstração de respostas emocionais^15^	
Clareza e fluência na expressão^15^	
Imitação de tom/entoação, expressões e posturas^15^	
Reações psicológicas realistas^15^	
Pontualidade e dedicação^15^	Integridade Profissional^21^
Integridade profissional em simulação clínica^12^	
Participação em simulações interprofissionais^12^	Simulação Interprofissional^26^
Conteúdo da avaliação^15^	Aprendizagem e Desempenho^27^
Critérios de pontuação para cada indicador^15^	
Melhoria no desempenho clínico pós-simulação clínica^12^	
Índice NPS* de satisfação dos alunos/profissionais com a simulação clínica^12^	
Efetividade da reflexão e aprendizado^12^	
Realismo Conseguido (Objetivo)^13^ ^-^ ^14^	
Realismo Percebido (Subjetivo)^13^ ^-^ ^14^	
Precisão da avaliação como avaliador^15^	
Atenção ao desempenho dos estudantes^15^	
Tempo médio de *debriefing* em simulação clínica^12^	O Processo do *Debriefing* ^25^
*Debriefing* com foco em reflexão e *soft skills* ^17^	
Melhoria na competência clínica^12^	Resultados e objetivos^28^
Eficácia na conquista de objetivos de aprendizado em simulações clínicas^12^	
Média de treinamentos realizados por profissionais simulacionistas para capacitação em simulação clínica^12^	Desenvolvimento profissional^23^

*Índice *Net Promoter Score* (NPS)

### Indicadores de estrutura

Nesta categoria, foram mapeados 27 indicadores, fundamentados principalmente nos padrões de operações, integridade profissional e *design* da simulação^20^ ^-^ ^22^. O padrão *“Design* da Simulação” foi o mais expressivo, concentrando métricas voltadas à tecnologia, fidelidade ambiental e realismo cenográfico^12^ ^-^ ^14^ ^,^ ^16^ ^-^ ^17^. Destaca-se que a maioria desses indicadores busca assegurar que a infraestrutura física e tecnológica esteja alinhada aos objetivos pedagógicos, com ênfase na preparação do ambiente e da equipe de facilitação.

### Indicadores de processo

Essa categoria abrange 45 indicadores subdivididos nos seguintes padrões de melhores práticas: *design* da simulação; pré-*briefing* (preparação e *briefing*); o processo do *debriefing*; facilitação; integridade profissional; simulação interprofissional continuada e avaliação da aprendizagem e do desempenho^19^ ^,^ ^21^ ^-^ ^22^ ^,^ ^24^ ^-^ ^27^.

A análise revela uma forte concentração em aspectos da prática pedagógica, como o alinhamento curricular, a condução do *debriefing* e a performance do participante simulado^13^ ^-^ ^17^. Observou-se que os indicadores de processo priorizam a interação entre facilitador e aluno, a consistência das simulações e a avaliação da prontidão do estudante, refletindo uma preocupação com a integridade da experiência de aprendizagem.

### Indicadores de resultado

Foram identificados 12 indicadores nesta dimensão, com foco predominante no padrão de avaliação da aprendizagem e desempenho^27^. Os pontos mais relevantes incluem a mensuração da melhoria no desempenho clínico pós-simulação, o índice de satisfação (NPS) dos usuários e a efetividade da reflexão alcançada no *debriefing* ^12^. Nota-se que os resultados são majoritariamente voltados para o desfecho do aprendizado e para a capacitação contínua da equipe responsável pela simulação.

## Discussão

Esta revisão de escopo teve como objetivo mapear na literatura os indicadores de qualidade para gestão de centros/laboratórios de simulação clínica. A análise dos seis estudos incluídos permitiu identificar um conjunto diversificado de indicadores, os quais foram categorizados segundo a tríade clássica de Donabedian - Estrutura, Processo e Resultado^7^ -e correlacionados com os padrões de melhores práticas da INACSL^18^.

Ao analisar os estudos incluídos nesta revisão, destaca-se que parte das publicações apresenta uma continuidade metodológica e conceitual. As pesquisas desenvolvidas mostraram sua natureza aplicada, ao utilizarem indicadores previamente definidos^16^ ^-^ ^17^, além de aprofundarem e operacionalizarem os conceitos propostos, o que indica uma trajetória de amadurecimento teórico sobre a fidelidade na simulação clínica^13^ ^-^ ^14^.

A comparação entre os indicadores mapeados neste estudo evidencia uma lacuna substancial no que se refere à abordagem dos processos gerenciais dos centros/laboratórios de simulação clínica. Apesar de elementos como fidelidade, realismo, *design* pedagógico e estrutura física dos ambientes simulados serem importantes e amplamente contemplados nos estudos selecionados^13^ ^-^ ^17^, observa-se que a maioria das produções prioriza aspectos vinculados à prática pedagógica da simulação, deixando de abordar adequadamente os componentes administrativos e operacionais que sustentam o funcionamento dos centros/laboratórios de simulação clínica.

Dessa forma, evidenciou-se que, apesar do grande número de indicadores identificados, estes não representam um conjunto aplicável diretamente à avaliação plena dos processos gerenciais dos centros/laboratórios de simulação clínica, objeto do presente estudo. Acredita-se que questões como planejamento, organização de recursos, capacitação da equipe, gerenciamento de insumos, manutenção de equipamentos, definição de metas e monitoramento contínuo ainda são abordadas de forma incipiente em artigos científicos. Essa ausência pode comprometer a capacidade de aferição da qualidade dos processos gerenciais, limitando o uso dos indicadores como ferramenta de gestão estratégica.

Um estudo elaborou e validou, com auxílio de especialistas, diversos indicadores de qualidade elencados em quatro domínios, denominados respectivamente como “princípios pedagógicos”, “preparação do estudante”, “fidelidade” e “*debriefing*”^16^. Esses indicadores são adequados para mensurar a qualidade do aspecto pedagógico e a implementação da metodologia de acordo com recomendações abordadas pelos padrões da INACSL^18^. Entretanto, compreende-se que ainda abordam de forma pouco objetiva a gestão de centros/laboratórios de simulação clínica. 

Os indicadores de gestão representam não apenas uma exigência para a mensuração do desempenho, mas também uma ferramenta estratégica para a manutenção do programa de simulação. Além disso, os processos de acreditação de programas de simulação têm exigido cada vez mais a apresentação de métricas consolidadas que atestem a efetividade das práticas pedagógicas e gerenciais^29^.

O processo de acreditação da *Society for Simulation in Healthcare* (SSH) exige que os centros/laboratórios de simulação clínica demonstrem excelência em áreas como governança, sustentabilidade financeira, recursos humanos e gestão de instalações^30^.

Por outro lado, uma pesquisa apresentou indicadores nos domínios da “avaliação do estudante como paciente simulado” e da ‟avaliação enquanto avaliador”, contemplando métricas como ‟Consistência em cada performance”, ‟Desenvolvimento do enredo do roteiro”, ‟Imitação de tom/entoação, expressões e posturas” e ‟Clareza e fluência na expressão”^15^. Embora esses indicadores não tratem especificamente dos processos gerenciais de centros/laboratórios de simulação clínica, acredita-se que podem ser considerados pelos gestores como Indicadores-Chave de Desempenho (*Key Performance Indicators* - KPIs), a depender dos objetivos institucionais e do estágio de desenvolvimento do programa de simulação.

Um KPI na área da saúde corresponde a uma métrica objetiva empregada para acompanhar, avaliar, aprimorar, controlar e promover mudanças nos processos assistenciais, com o propósito de assegurar a eficácia, a qualidade e a eficiência dos serviços, bem como elevar a satisfação de pacientes e profissionais de saúde^31^. Entende-se que os KPIs em programas de simulação clínica devem auxiliar os gestores na análise de dados de treinamento e na organização das atividades do centro/laboratório de simulação clínica.

Seguindo essa linha de raciocínio, identificou-se um conjunto de indicadores voltados ao planejamento e à estruturação das práticas de simulação, organizados em domínios como “fidelidade conceitual”, “fidelidade física”, “fidelidade emocional”, “participante simulado”, “cenografia” e “simulador”. Dentre os diversos indicadores destacados pelos autores, predominam aqueles relacionados ao realismo cenográfico (como mobiliário fixo, efeitos sonoros, odores, iluminação), ao realismo do simulador humanoide (características físicas, vestimentas, tato, voz, *moulage*), à interpretação do participante simulado (abrangendo caracterização conceitual e emocional, improvisação e interação com o aluno), além dos aspectos de realismo alcançado e realismo percebido^13^ ^-^ ^14^.

Embora tais métricas sejam fundamentais para avaliar o realismo, podendo auxiliar a efetividade do ensino baseado em simulação e verificar o alcance dos objetivos educacionais propostos, entende-se que não contemplam diretamente os processos gerenciais dos centros/laboratórios de simulação clínica. Ainda assim, podem oferecer subsídios relevantes que permitam extrapolações ou reflexões aplicáveis à gestão desses ambientes.

Nesse contexto, pesquisadores ressaltam o papel dos centros/laboratórios de simulação clínica como estruturas que articulam a universidade, o sistema de saúde e a gestão pública. Os autores reforçam a relevância da integração entre gestão, ensino e serviço, bem como a necessidade de avaliar, com base em evidências, o impacto do uso da simulação na formação profissional e na qualidade da assistência em saúde^32^.

Dessa forma, compreende-se que mesmo os indicadores que não abordam diretamente os aspectos gerenciais dos centros/laboratórios de simulação clínica podem subsidiar estratégias de gestão nesses ambientes. O estudo ainda propõe a seguinte indagação: “quais indicadores podem auxiliar na averiguação dos efeitos da simulação sobre a formação e os serviços?”, evidenciando, assim, uma lacuna na literatura que merece ser explorada^32^.

Este achado parece apontar para uma dicotomia entre indicadores de ensino-aprendizagem e gerenciais. Na verdade, embora sejam complementares, acredita-se que os processos de planejamento, operação, sustentabilidade, logística, capacitação da equipe, manutenção de equipamentos e análise de resultados institucionais demandam indicadores próprios, que vão além da avaliação do desempenho dos discentes.

Alguns estudos apresentaram uma abordagem mais alinhada ao escopo desta revisão, ao propor indicadores organizados nos domínios de “infraestrutura”, “recursos tecnológicos”, “princípios pedagógicos” e “preparação dos envolvidos”^16^ ^-^ ^17^. Com base nessa estrutura, entende-se que indicadores como a “tecnologia alinhada aos objetivos”, “Equipamentos e prontuários realistas”, “Matriz curricular da simulação integrada”, “Materiais preparatórios disponíveis”, bem como “Objetivos de aprendizagem guiam o *design* da simulação”^16^ ^-^ ^17^, podem ser considerados, ainda que indiretamente, como componentes de processos gerenciais em centros/laboratórios de simulação clínica, contribuindo para a qualificação da gestão e para o alinhamento entre práticas pedagógicas e recursos institucionais.

Diante disso, é pertinente destacar que os Padrões de Melhores Práticas em Simulação em Saúde estabelecem diretrizes específicas para a operação de centros/laboratórios de simulação clínica. Acredita-se que tais diretrizes podem servir como referência para a formulação de indicadores de qualidade, abrangendo tanto a dimensão pedagógica quanto os aspectos relacionados à gestão desses espaços^20^.

O padrão “Operações”, estabelecido pela INACSL, destaca que “todos os programas de educação baseados em simulação requerem sistemas e infraestrutura para apoiar e manter as operações”^20^. Essa diretriz evidencia a importância de práticas organizacionais bem definidas para garantir a sustentabilidade e a qualidade das atividades desenvolvidas nos centros/laboratórios de simulação clínica.

Neste sentido, um estudo apresentou uma proposta de indicadores voltados diretamente à gestão de centros/laboratórios de simulação clínica, estruturados nos domínios de “estrutura”, “processo” e “resultados”. Entre os exemplos, é possível citar a “taxa de disponibilidade de recursos de simulação”, a “taxa de equipamentos de simulação avariados”, a “avaliação da segurança do ambiente de simulação”, “melhoria no desempenho clínico pós-simulação clínica”, além da “satisfação dos alunos/profissionais com a simulação clínica”^12^. No entanto, tais indicadores ainda demandam maior refinamento metodológico e validação por especialistas da área.

Considera-se que a construção e validação de indicadores consistentes e aplicáveis pode contribuir para a gestão dos centros/laboratórios de simulação clínica, favorecendo o controle organizacional, a melhoria contínua, a transparência institucional e o alinhamento com referenciais internacionais, como os propostos pela INACSL^18^. No entanto, a efetiva implementação desses indicadores pode enfrentar obstáculos, especialmente em função das particularidades e limitações de cada instituição.

Neste contexto, pode ainda ser ressaltado que a elaboração de um conjunto de políticas e procedimentos direcionados ao funcionamento dos centros/laboratórios de simulação clínica representa um desafio, demandando tempo e investimento de recursos. Entre os principais obstáculos estão: a carência de sistemas de informação eficientes para a coleta e análise de dados; a resistência institucional à adoção de mudanças e à implementação de novos mecanismos de monitoramento; a ausência de parâmetros comparativos consolidados (*benchmarks*) para avaliação de desempenho; as dificuldades em mensurar o impacto das práticas de gestão nos resultados educacionais; e a disparidade nos recursos disponíveis entre as instituições^29^.

Superar esses desafios demanda estratégias colaborativas e flexíveis, que considerem as especificidades de cada realidade institucional sobre a articulação entre gestão, ensino e serviço^29^ ^,^ ^31^. Nesse sentido, entende-se que, após a definição dos processos gerenciais - preferencialmente com base em *benchmarks* nacionais e internacionais -, é fundamental promover estudos relacionados à validação de indicadores específicos para a gestão de centros/laboratórios de simulação clínica.

Dentre os seis estudos incluídos na amostra desta pesquisa, observou-se que somente um se propôs a abordar especificamente a elaboração de indicadores para a gestão de centros/laboratórios de simulação clínica^12^. Porém, esses indicadores carecem de validação por especialistas.

Como limitações do estudo, destaca-se a incipiência da abordagem dos indicadores de qualidade em simulação clínica na literatura científica, especialmente no que se refere aos indicadores de processos gerenciais dos centros/laboratórios de simulação clínica. Além disso, os artigos analisados apresentam diversidade de contextos, objetivos e formatos metodológicos, o que dificultou a categorização e comparação direta dos indicadores.

Como implicações para o avanço do conhecimento científico na área de saúde e enfermagem, cabe destacar que o estudo oferece um panorama para que centros/laboratórios de simulação clínica e gestores em enfermagem possam estruturar critérios de avaliação dos programas de simulação baseados em indicadores, promovendo maior objetividade e padronização. Além disso, acredita-se que o fomento da discussão sobre a integração entre o planejamento pedagógico e a gestão em simulação pode embasar critérios institucionais, reforçando a cultura de qualidade e segurança no ensino da saúde.

## Conclusão

O estudo mapeou a literatura científica sobre os indicadores de processos gerenciais em centros/laboratórios de simulação clínica, norteado pela pergunta de pesquisa: Quais indicadores de qualidade são necessários para a gestão de centros/laboratórios de simulação clínica?

Os resultados oferecem subsídios para que centros/laboratórios de simulação clínica possam estruturar critérios de avaliação baseados em indicadores, promover maior eficiência e alinhamento entre gestão e práticas educacionais e melhorar a comunicação. Não obstante, evidenciou-se uma lacuna significativa relacionada à definição e validação de indicadores, em particular, à gestão dos centros/laboratórios de simulação clínica. Constatou-se que, embora a temática seja fundamental para a sustentabilidade e a efetividade dos espaços formativos, a maioria das publicações concentra-se em aspectos pedagógicos da simulação, tais como realismo, *design* instrucional e avaliação discente, dedicando pouca atenção às dimensões administrativas e operacionais. 

Diante desse cenário, destaca-se a necessidade de incentivar pesquisas metodológicas e aplicadas que possibilitem o desenvolvimento de indicadores específicos, validados e alinhados a padrões internacionais de qualidade e acreditação. Acredita-se que a construção de um sistema de indicadores voltado aos processos gerenciais pode contribuir para a promoção da transparência, o aumento da eficiência e o fortalecimento do alinhamento estratégico entre as práticas pedagógicas e a gestão institucional dos centros/laboratórios de simulação clínica. 

Recomenda-se, portanto, que investigações futuras avancem na proposição e validação de indicadores de processos gerenciais dos centros/laboratórios de simulação clínica, considerando suas particularidades e os desafios inerentes à sua operação, com o objetivo de qualificar continuamente os espaços de ensino-aprendizagem em saúde.

Sugere-se ainda a realização de estudos quantitativos de avaliação da qualidade em simulação clínica, cujos resultados expostos podem facilitar a otimização de recursos, a tomada de decisões e o planejamento de práticas gerenciais concordantes com as reais necessidades do setor saúde.

## Data Availability

Todos os dados gerados ou analisados durante este estudo estão incluídos neste artigo publicado.

# Translation: Indicadores de calidad para la gestión de centros y laboratorios de simulación clínica: revisión de alcance

**DOI:** 10.1590/1518-8345.8215.4905

**Authors:** Isaac Sebastião Nunes Santos, Cláudio José de Souza, Rodrigo Leite Hipolito, Márcia Farias de Oliveira, Carolina Baptista Ribeiro, André da Silva Brites

**Affiliations:** 1 Universidade Federal Fluminense, Escola de Enfermagem Aurora de Afonso Costa, Niterói, RJ, Brasil.; 2 Hospital Universitário Antônio Pedro, Centro de Terapia Intensiva, Niterói, RJ, Brasil.

**Keywords:** Entrenamiento Simulado, Indicadores de Gestión, Indicadores de Calidad de la Atención de Salud, Garantía de la Calidad de Atención de Salud, Organización y Administración, Gestión en Salud

## Abstract

**Objetivo:**

mapear em la literatura los indicadores de calidad para la gestión de centros y laboratorios de simulación clínica.

**Método:**

revisión de alcance realizada conforme a las directrices del JBI e la extensión *Preferred Reporting Items for Systematic Reviews and Meta-Analyses for Scoping Reviews*. Se realizaron búsquedas sobre indicadores de calidad para la gestión de centros/laboratorios de simulación clínica, publicados desde 1990, en cuatro fuentes de información y en Google *Scholar*, sin restricciones de método, idioma u origen. Los datos se extrajeron tras agrupaciones temáticas y se categorizaron según la tríada Estructura, Proceso y Resultado, recomendada por Donabedian, correlacionándolos con los estándares de buenas prácticas de la *International Nursing Association for Clinical Simulation and Learning*.

**Resultados:**

de los seis estudios seleccionados, entre 2013 y 2024 se mapearon ochenta y cuatro indicadores de calidad. A pesar del alto número de indicadores, no son completamente aplicables a la evaluación de los procesos de gestión de los centros/laboratorios de simulación clínica. La planificación, organización de recursos, formación de equipos, gestión de insumos, mantenimiento de equipos y monitorización continua aún están en fase incipiente en la literatura.

**Conclusión:**

se recomienda que futuras investigaciones propongan y validen indicadores de los procesos de gestión de los centros/laboratorios de simulación clínica, considerando sus particularidades y desafíos operativos, con el objetivo de la cualificación continua de los espacios de enseñanza-aprendizaje en salud.

## Destacados

**(1)** Se identificaron 84 indicadores de calidad para la gestión de centros de simulación clínica. **(2)** Los indicadores de evaluación aún no son del todo aplicables a los procesos de gestión. **(3)** Predominio de los indicadores pedagógicos sobre los de gestión. **(4)** Subvenciones para la estructuración de indicadores específicos para la evaluación de la gestión. **(5)** Necesidad de validar los indicadores de gestión en los centros de simulación clínica.

## Introducción

La simulación clínica se ha consolidado como una herramienta para la enseñanza y formación de estudiantes y profesionales de la salud. Es una técnica que crea una situación o entorno simulado para permitir que los profesionales sanitarios experimenten la representación de un evento real, facilitando la práctica, el aprendizaje, la evaluación, la prueba o la comprensión de sistemas o acciones humanas. Además de los beneficios directos para la educación y formación de estudiantes/profesionales de la salud, la simulación clínica también permite optimizar los sistemas de salud, mejorando la atención proporcionada y contribuyendo a la seguridad del paciente^1^.

El uso de la simulación en la educación en salud comenzó en el siglo XVIII, con maniquíes anatómicos destinados a la enseñanza de obstetricia en Francia; Sin embargo, adquirió mayor impulso a partir de los años 50, con el desarrollo de simuladores electrónicos de baja y alta fidelidad capaces de reproducir funciones fisiológicas, responder a medicamentos y enseñar la gestión básica de las vías respiratorias^2^ ^-^ ^3^. En las décadas siguientes, los avances tecnológicos dieron lugar a simuladores cada vez más sofisticados, transformando la capacitación en salud y estimulando la creación de centros y laboratorios de simulación; sin embargo, el alto coste de equipos y *software* especializados sigue siendo un desafío para instituciones con recursos limitados^4^.

Dado este escenario, es necesario considerar que la implementación de un programa de simulación requiere una inversión significativa de recursos, personal y tiempo. Esto debe ser reconocido y considerado cuidadosamente antes del inicio del proyecto mediante un análisis de coste-efectividad basado en los posibles beneficios en la práctica clínica, así como en los resultados para los pacientes. Por tanto, la eficacia por sí sola no justifica grandes inversiones, y es esencial también analizar la eficiencia, es decir, cuánto valor se genera en relación con el coste empleado^5^.

Dada la expansión de los centros/laboratorios de simulación clínica en instituciones educativas y servicios de salud, es esencial que estos entornos se gestionen eficazmente, para garantizar su relevancia en el proceso educativo y su impacto positivo en la formación profesional. Para ello, es esencial que la gestión de estos espacios incorpore, entre otras estrategias, el desarrollo y uso de indicadores de calidad, capaces de monitorizar y evaluar su funcionamiento, rendimiento y contribución a la mejora de la enseñanza^6^.

El uso de indicadores de calidad se ha consolidado como una práctica recurrente en la administración de los servicios de salud, ya que permite tanto la evaluación de resultados como la mejora continua de las actividades pedagógicas y operativas^7^. En el contexto de los centros/laboratorios de simulación, se considera importante que estos indicadores abarquen elementos relacionados con la estructura, los procesos y los resultados, abarcando aspectos como la infraestructura disponible, la formación de instructores, las metodologías adoptadas y los efectos observados en el aprendizaje de estudiantes/profesionales de la salud.

Los indicadores de calidad, ya sean cualitativos o cuantitativos, son esenciales para apoyar la toma de decisiones estratégicas. Su selección y uso deben planificarse cuidadosamente, teniendo en cuenta las especificidades de los centros y laboratorios de simulación clínica, los requisitos relacionados con la acreditación y los objetivos estratégicos previamente definidos^8^.

Se cree que la creciente implementación de centros/laboratorios de simulación clínica exige una gestión eficiente y basada en la evidencia. Sin embargo, la literatura carece de estudios que aborden indicadores específicos dirigidos a los procesos de gestión de estos entornos. La ausencia de estos parámetros compromete la evaluación de la eficiencia y la mejora continua. Este estudio se justifica por la necesidad de mapear indicadores de calidad aplicables a la gestión de centros/laboratorios de simulación clínica, contribuyendo a su sostenibilidad, alineación estratégica y calificación de las prácticas educativas. En este contexto, el objetivo de este estudio fue mapear en la literatura los indicadores de calidad para la gestión de centros y laboratorios de simulación clínica.

## Método

### Tipo de revisión 

Esta revisión de alcance sigue las directrices y recomendaciones del manual del JBI y las directrices del *Preferred Reporting Items for Systematic Reviews and Meta-Analyses Extension for Scoping Reviews* (PRISMA-ScR)^9^ ^-^ ^10^.

Este formato de revisión permite mapear y recopilar una amplia variedad de materiales relacionados con un tema específico, ofreciendo una comprensión amplia del asunto. También permite considerar estudios con distintos niveles de evidencia y diferentes métodos, incluyendo fuentes de información y literatura gris. Sin embargo, no pretende realizar una evaluación crítica, ni analizar el riesgo de sesgo o jerarquización de los niveles de evidencia^9^.

Para garantizar la fiabilidad y equidad del proceso de revisión, el protocolo fue registrado en el *Open Science Framework* (https://doi.org/10.17605/OSF.IO/6AGBE).

### Criterios de elegibilidad

En esta revisión se incluyeron estudios originales cuyos objetos abordaron indicadores de calidad para la gestión de centros/laboratorios de simulación clínica. Se consideraron estudios publicados a partir de 1990, considerando que este fue el periodo de mayor desarrollo de la simulación clínica. No hubo restricciones respecto al tipo de diseño metodológico, idioma o país de origen de los estudios, incluyendo publicaciones que cumplieran con la pregunta de investigación y los criterios de inclusión definidos en base a la estrategia mnemónica Población-Contexto-Concepto (PCC), aplicada en las revisiones de alcance. Se excluyeron los estudios que no seguían el tema, los que no cumplían completamente con los componentes establecidos de la PCC y las publicaciones duplicadas entre las bases de datos.

Según las directrices del manual JBI, el mnemotécnico PCC debe seguir el estándar de referencia (Población, Concepto y Contexto) relevante para la definición del título claro y esta definición debe aparecer de forma idéntica en los criterios objetivos, de investigación y de inclusión. En este estudio, Población = centros/laboratorios de simulación clínica; Concepto = indicadores de calidad/gestión; Contexto = gestión y seguimiento de estos centros. Por tanto, la pregunta de revisión establecida fue: ¿Cuáles son los indicadores de calidad necesarios para la gestión de los centros/laboratorios de simulación clínica?

### Fuentes de información

La búsqueda de artículos se realizó en fuentes de información de la literatura científica, incluyendo el *Medical Literature Analysis and Retrieval System Online* (MEDLINE) a través de PubMed, el *Cumulative Index to Nursing and Allied Health Literature* (CINAHL), la *Latin American and Caribbean Literature on Health Sciences* (LILACS) y Scopus. Además, Google *Scholar* se utilizaba para buscar tesis y tesis (literatura gris), así como la lista de referencias.

### Estrategias de búsqueda

Las estrategias de búsqueda fueron desarrolladas por el autor principal, junto con su asesor y con el apoyo de un bibliotecario especializado en la construcción de estrategias de búsqueda para revisiones de alcance. Para garantizar la adecuación de los términos y fuentes, se celebró una consulta preliminar el 15 de agosto de 2024.

La estrategia de búsqueda se llevó a cabo asociando descriptores y términos sinónimos libres correspondientes a los elementos del acrónimo PCC, con el objetivo de identificar evidencia científica que respondiera a la pregunta de investigación de este estudio. Para la selección de términos, se consultaron vocabularios controlados en el área de salud, incluyendo DeCS (Descriptores de Ciencias de la Salud) y MeSH (*Medical Subject Headings*). 

Se incluyeron trabajos publicados a partir de 1990, considerando que este fue el periodo de mayor desarrollo de la simulación clínica. No se aplicaron filtros respecto al idioma ni al diseño del estudio, lo que garantizó una búsqueda amplia y exhaustiva. Además, las estrategias de búsqueda se desarrollaron siguiendo las directrices de la *Peer Review of Electronic Search Strategies* (PRESS), asegurando la calidad y rigor del proceso.

Las estrategias de búsqueda se desarrollaron basándose en una búsqueda preliminar, seguida del análisis de los títulos, resúmenes y palabras clave de los estudios recuperados, lo que permitió refinar y seleccionar términos relacionados con indicadores de calidad en salud y formación basada en simulación. Para responder a la pregunta de revisión, se utilizaron combinaciones de los descriptores *“Quality Indicators, Health Care”* y *“Quality Assurance, Health Care”*, así como términos libres y descriptores controlados asociados con simulación clínica y entrenamiento en simulación. Las búsquedas se estructuraron utilizando operadores booleanos y se adaptaron a las especificidades técnicas de cada base de datos, incluyendo variaciones lingüísticas cuando eran relevantes, como se muestra en Figura 1.

**Figura 1:** Estrategias de búsqueda en las bases de datos buscadas. Niterói, RJ, Brasil, 2025

**Estrategias de búsqueda**	
**PubMed**	(Quality Indicators, Health Care[mj] OR Quality Assurance, Health Care[mj] OR Quality Indicator*[tiab] OR Health Metric*[tiab] OR Quality Metric*[tiab] OR Quality Assessment[tiab] OR Quality Assurance[tiab] OR Indicator*[ti] OR Metric*[ti]) AND (Simulation Training[mj] OR Simulation Training[tiab] OR Simulation-based Training[tiab] OR Realistic Simulat*[tiab] OR Interactive Training[tiab] OR Interactive Learning[tiab] OR Interactive Simulat*[tiab] OR Clinical Simulat*[tiab] OR Healthcare Simulat*[tiab] OR Patient Simulat*[tiab])
**Scopus**	TITLE-ABS(“Quality Indicator*” OR “Health Metric*” OR “Quality Metric*” OR “Quality Assessment” OR “Quality Assurance” OR Indicator* OR Metric*) AND TITLE(“Simulation Training” OR “Simulation-based Training” OR “Realistic Simulation” OR “Interactive Training” OR “Interactive Learning” OR “Interactive Simulation” OR “Clinical Simulation” OR “Healthcare Simulation” OR “Patient Simulation”)
**CINAHL**	(“Quality Indicators” OR “Health Metrics” OR “Quality Metrics” OR “Quality Assessment” OR “Quality Assurance” OR Indicator* OR Metric* OR Indicador*) AND (“Simulation Training” OR “Simulation-based Training” OR “Realistic Simulation” OR “Interactive Training” OR “Interactive Learning” OR “Interactive Simulation” OR “Clinical Simulation” OR “Healthcare Simulation” OR “Simulación Clínica” OR “Entrenamiento de Simulación” OR “Simulación Realista”)
**LILACS**	(“Quality Indicators” OR “Health Metrics” OR “Quality Metrics” OR “Quality Assessment” OR “Quality Assurance” OR Indicator* OR Metric* OR Indicador*) AND (“Simulation Training” OR “Simulation-based Training” OR “Realistic Simulation” OR “Interactive Training” OR “Interactive Learning” OR “Interactive Simulation” OR “Clinical Simulation” OR “Healthcare Simulation” OR “Simulación Clinica” OR “Entrenamiento Simulado” OR “Simulación Realista” OR “Aprendizado Simulado” OR “Simulación Clínica” OR “Entrenamiento Simulado” OR “Simulación Realista”)
**Google Scholar**	(“Quality Indicators” OR “Quality Metrics” OR “Quality Assurance”) AND (“Simulation Training” OR “Clinical Simulation” OR “Healthcare Simulation”)

### Selección de los estudios

Para la selección de los estudios, todas las referencias bibliográficas identificadas mediante estrategias de búsqueda en bases de datos y fuentes de datos se exportaron a Rayyan^®^, donde se eliminaron los duplicados. Se incluyeron estudios primarios que abordaban la gestión de centros/laboratorios de simulación clínica y sus indicadores.

Inicialmente, dos revisores leían de forma anónima e independiente los títulos y resúmenes para verificar la elegibilidad de los estudios en función de los criterios de inclusión definidos, asegurándose de que cumplirían con la propuesta del estudio.

En la segunda fase, los revisores leían completamente los artículos seleccionados para confirmar los criterios de inclusión. Los desacuerdos se resolvieron por consenso o con la presencia de un tercer investigador para su evaluación.

### Extracción de datos

El proceso de extracción de datos fue realizado por el equipo de investigadores, utilizando un instrumento de recogida de datos alineado con el mnemotécnico PCC y con la pregunta de revisión propuesta. Es importante destacar que este instrumento está proporcionado por el manual del JBI y puede adaptarse según las necesidades de los autores del estudio^9^ ^,^ ^11^.

Basándose en el mnemotécnico PCC y para responder a la pregunta de revisión, se desarrolló un instrumento de extracción de datos que consistía en un formulario estructurado en la plataforma Google *Forms* ^*©*^ para identificar y describir los siguientes elementos: título, objetivo, año de publicación, tipo de estudio, país, objeto de investigación, pregunta de investigación e indicadores de calidad que podrían aplicarse a la gestión de centros/laboratorios de simulación clínica, separándolos según las dimensiones, la estructura, los procesos y los resultados.

### Separación, resumen e informe de resultados

Se recogieron datos de estudios seleccionados, lo que pone de manifiesto la relación de estos estudios con indicadores de calidad para la gestión de centros o laboratorios de simulación. La extracción de datos permitió mapear, interpretar y analizar la extensión, naturaleza y distribución de los estudios incluidos en la revisión. A continuación, se llevó a cabo el agrupamiento temático y se categorizó según la tríada clásica Estructura, Proceso y Resultado recomendada por Donabedian^7^.

Para el estudio propuesto, el marco se refiere a los estándares de buenas prácticas en simulación: *prebriefing* (preparación y *briefing*); operaciones; integridad profesional; diseño de simulación; desarrollo profesional y facilitación. En el proceso, se analizan los siguientes: diseño de simulación; *prebriefing* (preparación y *briefing* “reunión”); el proceso de *debriefing* (“informe”); facilitación; integridad profesional; simulación interprofesional continua y evaluación del aprendizaje y el rendimiento. El resultado está relacionado con los estándares de evaluación del aprendizaje y el rendimiento, el proceso de *debriefing*, los resultados y objetivos, además del nivel de desarrollo profesional. 

La descripción estadística de los datos también se realizó utilizando el Planillas Google^©^, con el objetivo de ofrecer una visión general del conjunto de materiales. Todo el procedimiento para la recogida de datos y selección de estudios fue resumido y presentado en los resultados de esta revisión, utilizando el diagrama de flujo PRISMA-ScR^10^.

## Resultados

Se identificaron un total de 698 estudios a partir de la consulta de fuentes y repositorios de información relevantes. Tras eliminar los registros duplicados, quedaban 582 estudios para análisis posteriores. Estos estudios se evaluaron leyendo sus títulos y resúmenes, con el objetivo de excluir aquellos que no encajaban con el tema investigado. Este cribado resultó en la selección de 20 estudios para evaluación en texto completo. Tras la lectura completa de estos materiales, se excluyeron 17 estudios: 14 porque no coincidían con el tema de la revisión y tres porque no cumplían los criterios de inclusión predefinidos (Figura 2)

**Figura 2:** Diagrama de flujo PRISMA-ScR para la identificación de los estudios. Niterói, RJ, Brasil, 2025

Tras el proceso de selección de los artículos identificados en las bases de datos y repositorios, la muestra de esta revisión comprendió seis estudios (n = 06), publicados en el periodo de 2013 a 2024. De esta muestra final, tres estudios (n=03; 50%) se originaron a partir de búsquedas sistemáticas, mientras que tres (n=03; 50%) se identificaron a través de la lista de referencias. En cuanto al formato, cinco estudios (n=05; 83,33%) se presentaron como artículos científicos y (n=01; 16,67%) como disertación de Máster. El enfoque limitado al tema de la gestión en la simulación, especialmente en lo que respecta a los indicadores de calidad de los centros/laboratorios clínicos de simulación, se hizo evidente en el pequeño número de estudios encontrados, incluso tras la aplicación de un marco temporal exhaustivo.

La Figura 3 muestra los datos más relevantes de cada artículo según el autor, el año/país/idioma, el tipo y el objetivo del estudio.

En cuanto a la formación de los autores de los estudios mencionados, se observa que cuatro publicaciones fueron producidas por enfermeras y dos por cirujanos dentistas. Todos los estudios constituyen investigación primaria.

Los únicos recursos informativos que devolvieron títulos para revisión y análisis fueron PubMed (tres artículos) y Google Scholar (dos artículos y una tesis de máster). Los indicadores de calidad de estos estudios se organizaron y correlacionaron con los estándares de mejores prácticas de la *International Nursing Association for Clinical Simulation and Learning* (INACSL)^18^ ^)^ (Figura 4). A continuación, los indicadores se categorizaron según el modelo teórico de evaluación de la calidad de la salud propuesto por Donabedian, en las dimensiones estructura, proceso y resultado, ampliamente utilizado en el análisis de la calidad de los servicios de salud, lo que permitió un enfoque sistemático de los indicadores identificados.

**Figura 3:** Descripción de los estudios incluidos en la revisión de alcance según el autor, el año/país/idioma, el tipo y el objetivo del estudio. Niterói, RJ, Brasil, 2025

N	Autor	Año/País/ Idioma	Tipo de estudio	Objetivo
**1**	Santos, 2024^12^	2024 Brasil Portugués	Estudio cualitativo	Analizar las buenas prácticas reportadas por los gestores de enfermería en centros/laboratorios de simulación clínica.
**2**	Montanet, et al., 2023^13^	2023 España Inglés	Estudio metodológico	Presentar a la comunidad científica la metodología detrás de una nueva herramienta numérica y digital para medir objetivamente el realismo en la simulación clínica.
**3**	Montanet, et al., 2020^14^	2020 España Español	Teórico-conceptual metodológico	Validar formularios universales para medir la fidelidad de la simulación.
**4**	Yuan, 2020^15^	2020 China Inglés	Estudio descriptivo	Elaborar el contenido de formación del Paciente Simulado Estándar (PSE), además de explorar su validez y fiabilidad.
**5**	Kable, et al., 2017^16^	2017 Australia Inglés	Estudio observacional	Evaluar una serie de sesiones de simulación utilizando un cronograma de observación desarrollado a partir de indicadores de calidad basados en la evidencia y comparar las diferencias en la obtención de resultados de calidad en los centros participantes del estudio.
**6**	Arthur, et al., 2013^17^	2013 Australia Inglés	Estudio de validación	Desarrollar un conjunto de indicadores de calidad aplicables internacionalmente que puedan utilizarse para guiar el desarrollo, la implementación y la evaluación de experiencias de simulación en los planes de estudio de enfermería de grado.

**Figura 4:** Indicadores de calidad de simulación clínica según la tríada estructura, proceso y resultados de Donabedian^7^ y los estándares de mejores prácticas en simulación de la INACSL^18^. Niterói, RJ, Brasil, 2025

Indicadores de estructura	Estándar INACSL
Evaluación de la preparación del equipo de facilitación^12^	*Prebriefing* ^19^
Tasa de disponibilidad de los recursos de simulación^12^	Operaciones^20^
Tasa de equipamientos de simulación averiados^12^	
Evaluación de la seguridad del entorno de simulación^12^	Integridad Profesional^21^
Índice de diversidad y fidelidad de escenarios de simulación clínica^12^	Diseño de la Simulación^22^
Tecnología alineada con los objetivos^16^ ^-^ ^17^	
Fidelidad ambiental alineada con los objetivos^16^ ^-^ ^17^	
Equipamientos y historiales médicos realista^s(16-17^)	
Caracterización Física del Participante Simulado^13^ ^-^ ^14^	
Realismo Escenográfico: Mobiliario Fijo^13^ ^-^ ^14^	
Realismo Escenográfico: Elementos Portátiles No Electrónicos^13^ ^-^ ^14^	
Realismo Escenográfico: Elementos Portátiles Electrónicos^13^ ^-^ ^14^	
Realismo Escenográfico: Elementos Sonoros^13^ ^-^ ^14^	
Realismo Escenográfico: Olores^13^ ^-^ ^14^	
Realismo Escenográfico: Material Sanitario Fungible^13^ ^-^ ^14^	
Realismo Escenográfico: Material Sanitario No Fungible^13^ ^-^ ^14^	
Realismo Escenográfico: Iluminación^13^ ^-^ ^14^	
Realismo del Simulador Humanoide: Características Físicas Generales^13^ ^-^ ^14^	
Realismo del Simulador Humanoide: Vestimenta^13^ ^-^ ^14^	
Realismo del Simulador Humanoide: Signos Vitales y Efectos Visibles^13^ ^-^ ^14^	
Realismo del Simulador Humanoide: Efectos Sonoros^13^ ^-^ ^14^	
Realismo del Simulador Humanoide: Tacto^13^ ^-^ ^14^	
Realismo del Simulador Humanoide: Voz^13^ ^-^ ^14^	
Realismo del Simulador Humanoide: *Moulage* y Maquillaje^13^ ^-^ ^14^	
Familiaridad con el currículo y la tecnología^16^ ^-^ ^17^	
Equipo entrenado en simulación^16^ ^-^ ^17^	Desarrollo profesional^23^
Facilitadores con conocimientos clínicos y pedagógico^16^ ^-^ ^17^	Facilitación^24^
Alineación con el currículo y los objetivos del curso^16^ ^-^ ^17^	Diseño de la Simulación^22^
Matriz curricular de la simulación integrada^16^ ^-^ ^17^	
Escalamiento de experiencias con apoyo progresivo^16^ ^-^ ^17^	
Integración de la simulación en todas las disciplinas clínicas^16^ ^-^ ^17^	
Los objetivos de aprendizaje guían el diseño de la simulación^16^ ^-^ ^17^	
Conocimiento de la enfermedad: síntomas, signos, tratamiento^15^	
Consistencia en cada desempeño^15^	
Desarrollo de la trama del guion^15^	
Características del paciente^15^	
Exámenes físicos relevantes, así como exámenes de laboratorio e imágenes^15^	
Realismo de la Interpretación del Participante Simulado: Caracterización Conceptual^13^ ^-^ ^14^	
Realismo de la Interpretación del Participante Simulado: Caracterización Emocional^13^ ^-^ ^14^	
Realismo de la Interpretación del Participante Simulado: Improvisación^13^ ^-^ ^14^	
Realismo de la Interpretación del Participante Simulado: Relación con el Estudiante^13^ ^-^ ^14^	
Realismo del Simulador Humanoide: Interacción con el Estudiante^13^ ^-^ ^14^	
Orientación estructurada al estudiante^16^ ^-^ ^17^	*Prebriefing* ^19^
Familiarización con maniquíes e equipamientos^16^ ^-^ ^17^	
*Briefing* sobre la estructura de la sesión^16^	
Claridad de los objetivos a los estudiantes^16^	
Materiales preparatorios disponibles^16^ ^-^ ^17^	
Entrenamiento previo de habilidades psicomotoras^16^	
Evaluación de la disposición para la simulación^12^	
*Debriefing* estructurado inmediato^16^ ^-^ ^17^	El Proceso del *Debriefing* ^25^
Reflexión y autoevaluación^16^	
Discusión de habilidades no técnicas^16^	
Comentarios sobre puntos fuertes^16^	
Comentarios sobre puntos débiles^16^	
*Feedback* combinado^16^	
Apoyo emocional tras rendimiento insatisfactorio^16^	
Tasa de efectividad de la facilitación^12^	Facilitación^24^
Cada actuación es consistente^15^	
La actuación es consistente con la trama de la pieza^15^	
Hacer frente a acontecimientos imprevistos^15^	
Memoria: historial médico y preocupaciones de salud^15^	
Demostración de síntomas^15^	
Demostración de signos^15^	
Demostración de respuestas emocionales^15^	
Claridad y fluidez en la expresión^15^	
Imitación del tono/entonación, expresiones y posturas^15^	
Reacciones psicológicas realistas^15^	
Puntualidad y dedicación^15^	Integridad Profesional^21^
Integridad profesional en la simulación clínica^12^	
Participación en simulaciones interprofesionales^12^	Simulación Interprofesional^26^
Contenido de la evaluación^15^	Aprendizaje y Rendimiento^27^
Criterios de puntuación para cada indicador^15^	
Mejora en el rendimiento clínico tras la simulación clínica^12^	
Índice NPS* de satisfacción de los estudiantes/profesionales con la simulación clínica^12^	
Efectividad de la reflexión y aprendizaje^12^	
Realismo Alcanzado (Objetivo)^13^ ^-^ ^14^	
Realismo Percibido (Subjetivo)^13^ ^-^ ^14^	
Exactitud de la evaluación como evaluador^15^	
Atención al rendimiento de los estudiantes^15^	
Tiempo medio de *debriefing* en simulación clínica^12^	El Proceso del *Debriefing* ^25^
*Debriefing* con enfoque en la reflexión y *soft skills* ^17^	
Mejora en la competencia clínica^12^	Resultados y objetivos^28^
Eficacia para alcanzar los objetivos de aprendizaje en simulaciones clínicas^12^	
Media de entrenamientos realizados por profesionales de simulación para capacitación en simulación clínica^12^	Desarrollo profesional^23^

*Índice *Net Promoter Score* (NPS)

### Indicadores de estructura

En esta categoría, se cartografiaron 27 indicadores, basados principalmente en los estándares de operaciones, integridad profesional y diseño de simulación^20^ ^-^ ^22^. El patrón *“*Diseño de Simulación” fue el más expresivo, concentrando métricas centradas en tecnología, fidelidad ambiental y realismo escenográfico^12^ ^-^ ^14^ ^,^ ^16^ ^-^ ^17^. Cabe destacar que la mayoría de estos indicadores buscan asegurar que la infraestructura física y tecnológica esté alineada con los objetivos pedagógicos, con énfasis en la preparación del entorno y el equipo de facilitación.

### Indicadores de proceso

Esta categoría abarca 45 indicadores subdivididos en los siguientes estándares de buenas prácticas: diseño de simulación; *prebriefing* (preparación y *briefing*); el proceso de *debriefing*; facilitación; integridad profesional; simulación y evaluación interprofesional continua del aprendizaje y el rendimiento^19^ ^,^ ^21^ ^-^ ^22^ ^,^ ^24^ ^-^ ^27^.

El análisis revela una fuerte concentración en aspectos de la práctica pedagógica, como la alineación curricular, la realización de informes y el desempeño del participante simulado^13^ ^-^ ^17^. Se observó que los indicadores del proceso priorizan la interacción entre facilitador y estudiante, la coherencia de las simulaciones y la evaluación de la preparación del estudiante, reflejando una preocupación por la integridad de la experiencia de aprendizaje.

### Indicadores de resultado

Se identificaron 12 indicadores en esta dimensión, con un enfoque predominante en el estándar de evaluación del aprendizaje y el rendimiento^27^. Los puntos más relevantes incluyen la medición de la mejora en el rendimiento clínico tras la simulación, el índice de satisfacción (NPS) de los usuarios y la efectividad de la reflexión lograda en el *debriefing* ^12^. Cabe señalar que los resultados se centran principalmente en el resultado del aprendizaje y en la capacitación continua del equipo de simulación.

## Discusión

Esta revisión de alcance tuvo como objetivo mapear en la literatura los indicadores de calidad para la gestión de centros/laboratorios de simulación clínica. El análisis de los seis estudios incluidos permitió identificar un conjunto diverso de indicadores, que se categorizaron según la tríada clásica de Donabedian - Estructura, Proceso y Resultado^7^ - y correlacionados con los estándares de mejores prácticas de INACSL^18^.

Al analizar los estudios incluidos en esta revisión, cabe destacar que parte de las publicaciones presentan una continuidad metodológica y conceptual. Los estudios desarrollados demuestran su naturaleza aplicada, utilizando indicadores previamente definidos^16^ ^-^ ^17^, además de profundizar y operacionalizar los conceptos propuestos, lo que indica una trayectoria de madurez teórica respecto a la fidelidad en la simulación clínica^13^ ^-^ ^14^.

La comparación entre los indicadores mapeados en este estudio muestra una brecha sustancial en el enfoque de los procesos de gestión de los centros/laboratorios de simulación clínica. Aunque elementos como la fidelidad, el realismo, el diseño pedagógico y la estructura física de los entornos simulados son importantes y ampliamente contemplados en los estudios seleccionados^13^ ^-^ ^17^, se observa que la mayoría de las producciones priorizan aspectos relacionados con la práctica pedagógica de la simulación, sin abordar adecuadamente los componentes administrativos y operativos que apoyan el funcionamiento de los centros/laboratorios de simulación clínica.

Así, se evidenció que, a pesar del gran número de indicadores identificados, no representan un conjunto directamente aplicable a la evaluación completa de los procesos de gestión de los centros/laboratorios de simulación clínica, objeto de este estudio. Se considera que cuestiones como la planificación, organización de recursos, formación de equipos, gestión de entradas, mantenimiento de equipos, establecimiento de objetivos y monitorización continua aún se abordan de forma incipiente en los artículos científicos. Esta ausencia puede comprometer la capacidad de medir la calidad de los procesos de gestión, limitando el uso de indicadores como herramienta de gestión estratégica.

Un estudio elaboró y validó, con la ayuda de especialistas, varios indicadores de calidad listados en cuatro ámbitos, denominados respectivamente “principios pedagógicos”, “preparación del estudiante”, “fidelidad” y “*debriefing*”^16^. Estos indicadores son adecuados para medir la calidad del aspecto pedagógico y la implementación de la metodología según las recomendaciones abordadas por los estándares INACSL^18^. Sin embargo, se entiende que no abordan objetivamente la gestión de los centros/laboratorios de simulación clínica.

Los indicadores de gestión representan no solo un requisito para medir el rendimiento, sino también una herramienta estratégica para mantener el programa de simulación. Además, los procesos de acreditación de los programas de simulación han exigido cada vez más la presentación de métricas consolidadas que atestiguen la eficacia de las prácticas pedagógicas y gerenciales^29^.

El proceso de acreditación de la *Society for Simulation in Healthcare* (SSH) exige que los centros/laboratorios de simulación clínica demuestren excelencia en áreas como gobernanza, sostenibilidad financiera, recursos humanos y gestión de instalaciones^30^.

Por otro lado, un estudio presentó indicadores en los ámbitos de “evaluación del estudiante como paciente simulado” y ‟evaluación como evaluador”, contemplando métricas como ‟Consistencia en cada interpretación”, ‟Desarrollo de la trama del guion”, ‟Imitación de tono/entonación, expresiones y posturas” y “Claridad y fluidez en la expresión”^15^. Aunque estos indicadores no abordan específicamente los procesos de gestión de los centros/laboratorios de simulación clínica, se cree que los directivos pueden considerarlos como Indicadores Clave de Rendimiento (*Key Performance Indicators* - KPIs), dependiendo de los objetivos institucionales y la etapa de desarrollo del programa de simulación.

Un KPI en el área de salud corresponde a una métrica objetiva utilizada para monitorizar, evaluar, mejorar, controlar y promover cambios en los procesos de atención, con el objetivo de garantizar la eficacia, calidad y eficiencia de los servicios, así como aumentar la satisfacción de los pacientes y los profesionales de la salud^31^. Se entiende que los KPIs en los programas de simulación clínica deben ayudar a los gestores en el análisis de datos de entrenamiento y en la organización de las actividades del centro/laboratorio de simulación clínica.

Siguiendo esta línea de razonamiento, se identificó un conjunto de indicadores orientados a la planificación y estructuración de las prácticas de simulación, organizados en dominios como “fidelidad conceptual”, “fidelidad física”, “fidelidad emocional”, “participante simulado”, “escenografía” y “simulador”. Entre los diversos indicadores destacados por los autores, predominan aquellos relacionados con el realismo escénico (como mobiliario fijo, efectos de sonido, olores, iluminación), el realismo del simulador humanoide (características físicas, vestimenta, tacto, voz, moldeo), a la interpretación del participante simulado (incluyendo caracterización conceptual y emocional, improvisación e interacción con el estudiante), además de los aspectos de realismo alcanzado y realismo percibido^13^ ^-^ ^14^.

Aunque tales métricas son fundamentales para evaluar el realismo y pueden ayudar a la eficacia de la enseñanza basada en simulación y verificar el logro de los objetivos educativos propuestos, se entiende que no contemplan directamente los procesos de gestión de los centros/laboratorios de simulación clínica. Aun así, pueden ofrecer subvenciones relevantes que permitan extrapolaciones o reflexiones aplicables a la gestión de estos entornos.

En este contexto, los investigadores enfatizan el papel de los centros/laboratorios de simulación clínica como estructuras que articulan la universidad, el sistema sanitario y la gestión pública. Los autores refuerzan la relevancia de la integración entre gestión, docencia y servicio, así como la necesidad de evaluar, basándose en la evidencia, el impacto del uso de la simulación en la formación profesional y en la calidad de la atención a la salud^32^.

Por tanto, se entiende que incluso los indicadores que no abordan directamente los aspectos gerenciales de los centros/laboratorios de simulación clínica pueden apoyar estrategias de gestión en estos entornos. El estudio también plantea la siguiente pregunta: “¿qué indicadores pueden ayudar a determinar los efectos de la simulación en la formación y los servicios?”, evidenciando así una laguna en la literatura que merece ser explorada^32^.

Este hallazgo parece señalar una dicotomía entre los indicadores de enseñanza-aprendizaje y los indicadores gerenciales. De hecho, aunque son complementarios, se cree que los procesos de planificación, operación, sostenibilidad, logística, formación de equipos, mantenimiento de equipos y análisis de resultados institucionales requieren indicadores propios que van más allá de la evaluación del rendimiento de los estudiantes.

Algunos estudios presentaron un enfoque más alineado con el alcance de esta revisión, proponiendo indicadores organizados en los dominios de “infraestructura”, “recursos tecnológicos”, “principios pedagógicos” y “preparación de los implicados”^16^ ^-^ ^17^. Basándose en esta estructura, se entiende que indicadores como “tecnología alineada con los objetivos”, “Equipos y registros médicos realistas”, la “matriz integrada de currículo de simulación”, “Materiales preparatorios disponibles”, así como “Los objetivos de aprendizaje guían el diseño de la simulación”^16^ ^-^ ^17^, pueden considerarse, aunque sea indirectamente, como componentes de los procesos de gestión en centros/laboratorios de simulación clínica. contribuyendo a la cualificación de la gestión y a la alineación entre las prácticas pedagógicas y los recursos institucionales.

En vista de esto, es pertinente destacar que los Estándares de Mejores Prácticas en Simulación en Salud establecen directrices específicas para el funcionamiento de los centros/laboratorios de simulación clínica. Se cree que dichas directrices pueden servir como referencia para la formulación de indicadores de calidad, cubriendo tanto la dimensión pedagógica como los aspectos relacionados con la gestión de estos espacios^20^.

El estándar “Operaciones”, establecido por la INACSL, destaca que “todos los programas de educación basados en simulación requieren sistemas e infraestructura para apoyar y mantener las operaciones”^20^. Esta directriz destaca la importancia de prácticas organizativas bien definidas para garantizar la sostenibilidad y calidad de las actividades desarrolladas en los centros/laboratorios de simulación clínica.

En este sentido, un estudio presentó una propuesta de indicadores dirigidos directamente a la gestión de centros/laboratorios de simulación clínica, estructurados en los dominios de “estructura”, “proceso” y “resultados”. Entre los ejemplos, se puede mencionar la “tasa de disponibilidad de recursos de simulación”, la “tasa de equipos de simulación dañados”, la “evaluación de la seguridad del entorno de simulación”, “mejora del rendimiento clínico tras la simulación clínica”, además de la “satisfacción de estudiantes/profesionales con la simulación clínica”^12^. Sin embargo, estos indicadores aún requieren un refinamiento y validación metodológico adicional por parte de expertos en el area.

Se considera que la construcción y validación de indicadores consistentes y aplicables puede contribuir a la gestión de los centros/laboratorios de simulación clínica, favoreciendo el control organizativo, la mejora continua, la transparencia institucional y la alineación con referencias internacionales, como las propuestas por INACSL^18^. Sin embargo, la implementación efectiva de estos indicadores puede enfrentarse a obstáculos, especialmente debido a las particularidades y limitaciones de cada institución.

En este contexto, también cabe destacar que la elaboración de un conjunto de políticas y procedimientos orientados al funcionamiento de centros/laboratorios de simulación clínica representa un desafío que requiere tiempo e inversión de recursos. Entre los principales obstáculos se encuentran: la falta de sistemas de información eficientes para la recogida y análisis de datos; la resistencia institucional a la adopción de cambios y la implementación de nuevos mecanismos de supervisión; la ausencia de parámetros comparativos consolidados (*benchmarks*) para la evaluación del rendimiento; las dificultades para medir el impacto de las prácticas de gestión en los resultados educativos; y la disparidad en los recursos disponibles entre instituciones^29^.

Superar estos desafíos requiere estrategias colaborativas y flexibles que tengan en cuenta las especificidades de cada realidad institucional respecto a la articulación entre gestión, docencia y servicio^29^ ^,^ ^31^. En este sentido, se entiende que, tras definir los procesos de gestión -preferiblemente basados en referencias nacionales e internacionales- es esencial promover estudios destinados a validar indicadores específicos para la gestión de centros/laboratorios de simulación clínica.

De los seis estudios incluidos en la muestra de esta investigación, se observó que solo uno proponía abordar específicamente el desarrollo de indicadores destinados a la gestión de centros/laboratorios de simulación clínica^12^. Sin embargo, estos indicadores carecen de validación por parte de los expertos.

Como limitaciones del estudio, se destaca en la literatura científica la incipiencia del enfoque de indicadores de calidad en la simulación clínica, especialmente en lo que respecta a los indicadores de los procesos de gestión de los centros/laboratorios de simulación clínica. Además, los artículos analizados tienen una diversidad de contextos, objetivos y formatos metodológicos, lo que dificultaba categorizar y comparar directamente los indicadores.

Como implicaciones para el avance del conocimiento científico en el área de salud y enfermería, cabe destacar que el estudio ofrece una visión general para que los centros/laboratorios de simulación clínica y los responsables de enfermería estructuren criterios de evaluación para programas de simulación basándose en indicadores, promoviendo una mayor objetividad y estandarización. Además, se cree que fomentar el debate sobre la integración entre planificación pedagógica y gestión en simulación puede apoyar criterios institucionales, reforzando la cultura de calidad y seguridad en la educación en salud.

## Conclusión

El estudio mapeó la literatura científica sobre los indicadores de los procesos de gestión en centros/laboratorios de simulación clínica, guiado por la pregunta de investigación: ¿Qué indicadores de calidad son necesarios para la gestión de centros/laboratorios de simulación clínica?

Los resultados ofrecen subvenciones para que los centros/laboratorios de simulación clínica estructuren criterios de evaluación basándose en indicadores, promoviendo una mayor eficiencia y alineación entre la gestión y las prácticas educativas, colaborando en la comunicación de los resultados. No obstante, se evidenció una brecha significativa en cuanto a la definición y validación de indicadores específicamente dirigidos a la gestión de estos entornos. Se descubrió que, aunque el tema es fundamental para la sostenibilidad y eficacia de estos espacios de formación, la mayoría de las publicaciones se centran en aspectos pedagógicos de la simulación, como el realismo, el diseño instruccional y la evaluación del alumnado, prestando poca atención a las dimensiones administrativas y operativas.

En vista de este escenario, se destaca la necesidad de fomentar la investigación metodológica y aplicada que permita el desarrollo de indicadores específicos y validados, alineados con los estándares internacionales de calidad y acreditación. Se cree que la construcción de un sistema de indicadores centrados en los procesos de gestión puede contribuir a promover la transparencia, aumentar la eficiencia y fortalecer la alineación estratégica entre las prácticas pedagógicas y la gestión institucional de los centros/laboratorios de simulación clínica.

Por tanto, se recomienda que futuras investigaciones avancen en la propuesta y validación de indicadores de los procesos de gestión en estos entornos, considerando sus particularidades y los desafíos inherentes a su funcionamiento, con el objetivo de calificar continuamente los espacios de enseñanza-aprendizaje en salud.

También se sugiere que se realicen estudios cuantitativos para evaluar la calidad en la simulación clínica, cuyos resultados puedan facilitar la optimización de recursos, la toma de decisiones y la planificación de prácticas de gestión de acuerdo con las necesidades reales del sector de salud.

## Data Availability

Todos los datos generados o analizados durante este estudio están incluidos en este artículo publicado.
